# KRAS^G12D^-driven pentose phosphate pathway remodeling imparts a targetable vulnerability synergizing with MRTX1133 for durable remissions in PDAC

**DOI:** 10.1016/j.xcrm.2025.101966

**Published:** 2025-02-18

**Authors:** Xiangyan Jiang, Tao Wang, Bin Zhao, Haonan Sun, Yuman Dong, Yong Ma, Zhigang Li, Yuxia Wu, Keshen Wang, Xiaoying Guan, Bo Long, Long Qin, Wengui Shi, Lei Shi, Qichen He, Wenbo Liu, Mingdou Li, Lixia Xiao, Chengliang Zhou, Hui Sun, Jing Yang, Junhong Guan, Huinian Zhou, Zeyuan Yu, Zuoyi Jiao

**Affiliations:** 1 Department of General Surgery, Lanzhou University Second Hospital, Lanzhou 730000, China; 2 The Second Clinical Medical School, Lanzhou University, Lanzhou 730000, China; 3 Gansu Province High-Altitude High-Incidence Cancer Biobank, Lanzhou University Second Hospital, Lanzhou 730000, China; 4 Cuiying Biomedical Research Center, Lanzhou University Second Hospital, Lanzhou 730000, China; 5 State Key Laboratory of Applied Organic Chemistry, Lanzhou University, Lanzhou 730000, China; 6 Department of Pathology, Lanzhou University Second Hospital, Lanzhou 730000, China; 7 School of Public Health, Lanzhou University, Lanzhou 730000, China; 8 Radboud Institute for Molecular Life Sciences, Radboud University Medical Center, 6500 HB Nijmegen, the Netherlands

**Keywords:** KRAS^G12D^, MRTX1133, pentose phosphate pathway, metabolic reprogramming, pancreatic ductal adenocarcinoma

## Abstract

The KRAS^G12D^ inhibitor MRTX1133 shows the potential to revolutionize the treatment paradigm for pancreatic ductal adenocarcinoma (PDAC), yet presents challenges. Our findings indicate that KRAS^G12D^ remodels a pentose phosphate pathway (PPP)-dominant central carbon metabolism pattern, facilitating malignant progression and resistance to MRTX1133 in PDAC. Mechanistically, KRAS^G12D^ drives excessive degradation of p53 and glucose-6-phosphate dehydrogenase (G6PD)-mediated PPP reprogramming through retinoblastoma (Rb)/E2F1/p53 axis-regulated feedback loops that amplify ubiquitin-conjugating enzyme E2T (UBE2T) transcription. Genetic ablation or pharmacological inhibition of UBE2T significantly suppresses PDAC progression and potentiates MRTX1133 efficacy. Leveraging structure advantages of the UBE2T inhibitor pentagalloylglucose (PGG), we develop a self-assembling nano co-delivery system with F-127, PGG, and MRTX1133. This system enhances the efficacy of PGG and MRTX1133, achieving durable remissions (85% overall response rate) and long-term survival (100% progression-free survival) in patient-derived xenografts and spontaneous PDAC mice. This study reveals the role of KRAS^G12D^-preferred PPP reprogramming in MRTX1133 resistance and proposes a potentially therapeutic strategy for KRAS^G12D^-mutated PDAC.

## Highlights

• KRAS^G12D^-driven PPP remodeling promotes PDAC progression and MRTX1133 resistance
• KRAS^G12D^ promotes PPP reprogramming via Rb/E2F1/UBE2T/p53 feedback loops
• UBE2T confers progression and resistance to MRTX1133 in KRAS^G12D^-mutated PDAC
• Nano co-delivery of the UBE2T inhibitor and MRTX1133 achieves durable remissions

## Graphical abstract

**Figure:**
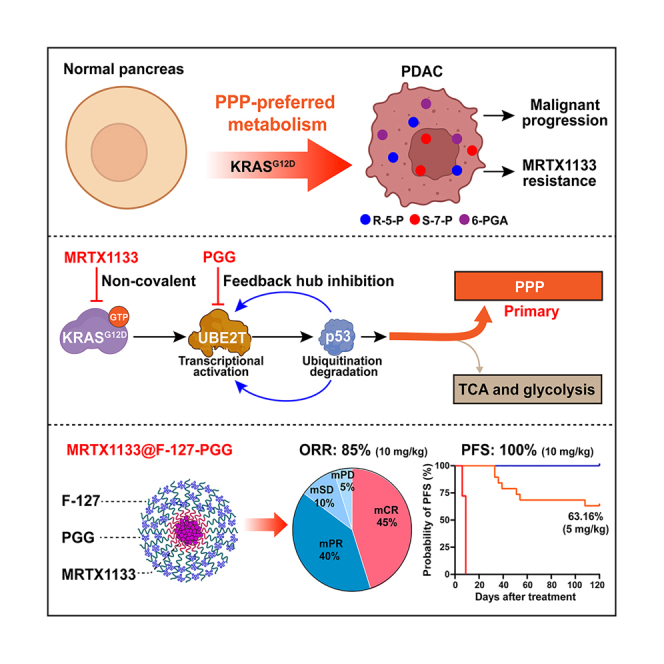

## Teaser

Jiang et al. uncover that KRAS^G12D^ drives a pentose phosphate pathway-dominant reprogramming of central carbon metabolism through UBE2T-mediated feedback mechanisms. They develop a nano co-delivery system combining F-127, the UBE2T inhibitor PGG, and the KRAS^G12D^ inhibitor MRTX1133, inducing significant tumor regression and durable therapeutic responses in KRAS^G12D^-mutant PDAC.

## Introduction

Pancreatic ductal adenocarcinoma (PDAC) is a highly lethal malignancy with an increasing incidence and a 5-year overall survival rate of only 12%.^1^ Although emerging immunotherapies and targeted treatments, such as pembrolizumab and olaparib, have been approved for PDAC treatment, their applicability is limited to rare molecular subtypes.^2^ KRAS^G12D^ is the most common oncogenic mutation in PDAC, as it is harbored by approximately 45% of patients.^3^ KRAS^G12D^ has been historically recognized as undruggable. Recently, MRTX1133, a non-covalent and selective KRAS^G12D^ inhibitor, has been developed.^4^ Functionally, MRTX1133 effectively suppresses oncogenic signaling pathways and exhibits potent antitumor efficacy in PDAC.^5^^,^^6^ In immunocompetent PDAC models, MRTX1133 reprograms the microenvironment and promotes immune cell-mediated killing.^7^^,^^8^^,^^9^ The available data suggest that MRTX1133 can alter the therapeutic paradigm of PDAC. However, resistance to KRAS inhibitors is a formidable challenge that needs to be addressed.^10^^,^^11^^,^^12^ The reactivation and feedback compensation of KRAS-associated vertical signaling pathways are critical factors contributing to MRTX1133 resistance.^13^^,^^14^ Therefore, a deeper understanding of the mechanisms that mediate resistance to MRTX1133 is imperative.

Metabolic reprogramming driven by KRAS^G12D^ mutations is a hallmark of high malignancy in PDAC.^15^^,^^16^ Oncogenic KRAS mutations remodel numerous metabolic programs, facilitating the malignant progression of PDAC.^17^^,^^18^^,^^19^^,^^20^ Central carbon metabolism, comprising glycolysis, the pentose phosphate pathway (PPP), and the tricarboxylic acid (TCA) cycle, serves as the primary source of energy and biomass supporting tumor cells.^21^ Hyperactivated central carbon metabolism contributes to malignant behaviors of PDAC, including precancerous lesions, progression, and treatment resistance.^22^^,^^23^^,^^24^^,^^25^ KRAS mutation-mediated signaling enhances cancer cell competitiveness and reduces therapeutic susceptibility by increasing glucose uptake and hexokinase activity, promoting pathways such as glycolysis, the TCA cycle, and PPP.^17^^,^^26^^,^^27^ However, the preference for a specific pattern of KRAS^G12D^-driven central carbon metabolism in PDAC and its detailed regulatory mechanisms are not well understood. Furthermore, the critical metabolic pathways that contribute to MRTX1133 resistance have not yet been identified. Therefore, investigating the regulatory mechanisms of KRAS^G12D^-driven central carbon metabolic reprogramming is crucial for developing treatments for KRAS^G12D^-mutant PDAC.

Here, we report that KRAS^G12D^ predominantly utilizes the PPP in central carbon metabolism, promoting malignant progression and MRTX1133 resistance in PDAC. We elucidate p53 ubiquitination-mediated ubiquitin-conjugating enzyme E2T (UBE2T) transcriptional feedback as an essential mechanism by which KRAS^G12D^ remodels the PPP. We also assessed the potential of UBE2T as a therapeutic target for PDAC with the KRAS^G12D^ mutation and evaluated the efficacy of targeting UBE2T with pentagalloylglucose (PGG) in overcoming resistance to MRTX1133. Furthermore, leveraging the polyphenolic structure advantage of PGG, interacted with pluronic F-127 to nanoencapsulate MRTX1133, creates a unique nanomedicine delivery system, demonstrating robust efficacy in PDAC with KRAS^G12D^ mutation.

## Results

### KRAS^G12D^ mutation remodels central carbon metabolism in PDAC, favoring PPP over glycolysis and the TCA cycle

**Figure 1:**
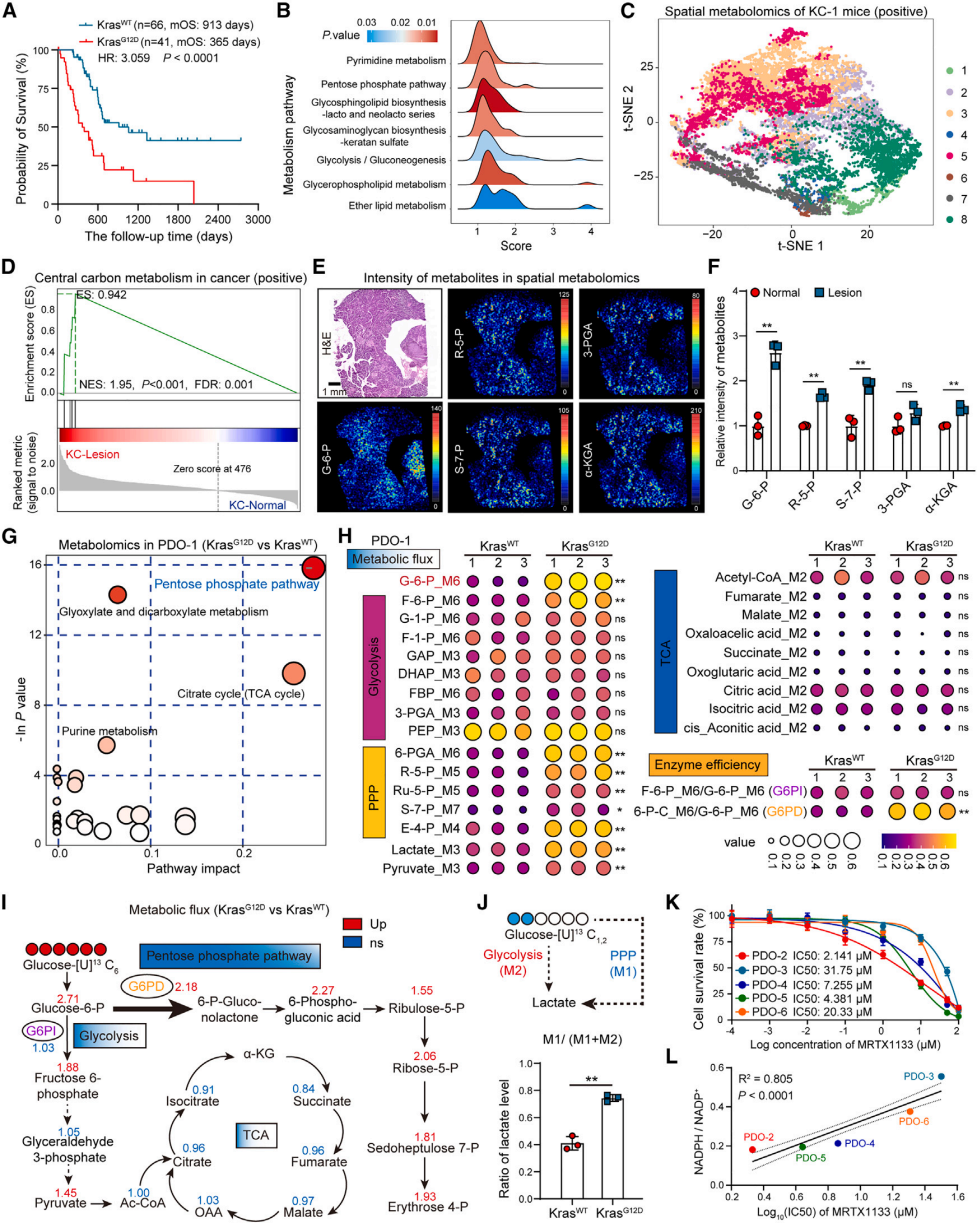
KRAS^G12D^ drives a PPP-dominant central carbon metabolism pattern in PDAC (A) Kaplan-Meier analysis with log-rank test showing OS for patients stratified by KRAS^WT^ and KRAS^G12D^ from TCGA database. (B) KEGG analysis for metabolic pathway using DEGs from patients with KRAS^WT^ or KRAS^G12D^ mutation in TCGA database. (C) t-distributed stochastic neighbor embedding (t-SNE) visualization of spatial metabolomics data from pancreatic tissues of KC mouse. (D) Gene set enrichment analysis (GSEA) of central carbon metabolism in normal pancreas and lesion based on spatial metabolomics data. (E and F) H&E staining and mass spectrometry imaging (MSI) of glucose-6-phosphate (G-6-P), ribose 5-phosphate (R-5-P), sedoheptulose 7-phosphate (S-7-P), 3-phosphoglyceric acid (3-PGA), and α-ketoglutaric acid (α-KGA), followed by statistical analysis (*n* = 3). (G) Metabolic pathway analysis of differential metabolites from targeted metabolomics on central carbon metabolism in KRAS^WT^ and KRAS^G12D^ PDO-1. (H) Heatmap displaying the indicated metabolite level from U-^13^C_6_-labeled metabolic flux analysis in KRAS^WT^ and KRAS^G12D^ PDO-1 (*n* = 3). Metabolite levels are represented by different sizes and colors of the indicated values. G-1-P, glucose 1-phosphate; F-1-P, fructose 1-phosphate; GAP, glyceraldehyde 3-phosphate; DHAP, dihydroxyacetone phosphate; FBP, fructose 1,6-bisphosphate; PEP, phosphoenolpyruvic acid; Ru-5-P, ribulose-5-phosphate; E-4-P, erythrose-4-phosphate. See Table S1. (I) Schematic example of U-^13^C_6_-labeled glucose metabolism in the glycolysis, PPP, and TCA cycle. Number represents the fold change of metabolites in KRAS^G12D^ compared to KRAS^WT^ PDO-1. (J) Ratio of lactate level (M1/M1+M2) from U-^13^C_1,2_-labeled metabolic flux analysis in WT and G12D-mutant PDO-1 (*n* = 3). (K) Sensitivity to MRTX1133 in PDO-2, 3, 4, 5, and 6 (*n* = 6). (L) Linear regression analysis shows the correlation of G6PD enzyme activity and MRTX1133 sensitivity. Mean ± SD, Student’s t test. ∗∗*p* < 0.01; ns, not significant. See also Figures S1–S3.

We analyzed data from clinical samples and the The Cancer Genome Atlas (TCGA) database to demonstrate that the oncogenic KRAS^G12D^ mutation is associated with a poor prognosis in PDAC (Figures 1A and S1A). The Kyoto Encyclopedia of Genes and Genomes (KEGG) pathway enrichment analysis of the differentially expressed genes (DEGs) between KRAS wild-type (WT) and G12D-mutant PDAC tissues from the TCGA database revealed abnormally upregulated metabolic pathways, including the PPP and glycolysis (Figures 1B and S1B).

Organoid lines stably overexpressing KRAS^G12D^ were established and used to conduct transcriptomic analyses on both WT and G12D patient-derived organoids (PDOs). The results revealed an abnormal upregulation of metabolic pathways, especially central carbon metabolism (Figures S1C–S1E). To determine the contribution of KRAS^G12D^-driven central carbon metabolism to the malignant progression of PDAC, a spontaneous PDAC model harboring *LSL-Kras*^*G12D/+*^ and *Pdx1-Cre* (KC) was utilized (Figures S2A and S2B), and spatial metabolomic analysis was conducted on pancreatic tissues. Dimensionality reduction clustering and differential metabolite analysis between normal and precancerous tissues revealed a significant upregulation of central carbon metabolism within lesion areas (Figures 1C, 1D, and S2C–S2H). Specifically, the concentrations of key metabolites of the central carbon metabolism pathway, including glucose-6-phosphate, ribose 5-phosphate, sedoheptulose 7-phosphate, and α-ketoglutaric acid, were significantly higher in the lesion areas compared with that in normal pancreatic tissues (Figures 1E and 1F).

To elucidate the preference of KRAS^G12D^ for regulating central carbon metabolism pathways in PDAC, targeted metabolomic analysis was performed on central carbon metabolism in both WT and G12D-mutant PDOs. Our findings suggested that KRAS^G12D^ exerted the most pronounced effect on the PPP (Figures 1G and S3A–S3F). ^13^C-labeled metabolic flux analysis using U-^13^C_6_ and U-^13^C_1,2_ glucose indicated that KRAS^G12D^ preferentially upregulated the PPP over glycolysis and the TCA cycle, directing the flow of glucose-6-phosphate predominantly toward the PPP, mediated by glucose-6-phosphate dehydrogenase (G6PD), the first and rate-limiting enzyme of the PPP, rather than through the glucose-6-phosphate isomerase-mediated glycolytic pathway (Figures 1H–1J; Table S1). Moreover, KRAS^G12D^-mutant PDOs exhibited higher G6PD enzyme activity compared with that of KRAS^WT^ PDOs (Figure S3G).

### Targeting G6PD-mediated PPP inhibits malignant progression and MRTX1133 resistance in PDAC with KRAS^G12D^ mutation

We assessed the correlation between MRTX1133 sensitivity and G6PD enzyme activity in KRAS^G12D^-mutant PDOs. Our data demonstrated that the half maximal inhibitory concentration (IC50) of MRTX1133 is positively correlated with G6PD enzyme activity (Figures 1K, 1L, S3H, and S3I). In KRAS^G12D^-mutant cell lines, we found that the intrinsically MRTX1133-resistant cell line PANC-1 exhibits higher G6PD enzyme activity compared to the sensitive cell line AsPC-1 (Figures S3J and S3K). Furthermore, we developed MRTX1133-acquired resistant AsPC-1 cell lines and observed that higher levels of resistance were associated with increased G6PD enzyme activity (Figures S3L and S3M). Treatment with RRx-001 rescued the sensitivity of PANC-1 and resistant AsPC-1 cells to MRTX1133 in a dose-dependent manner (Figures S3N and S3O).

**Figure 2:**
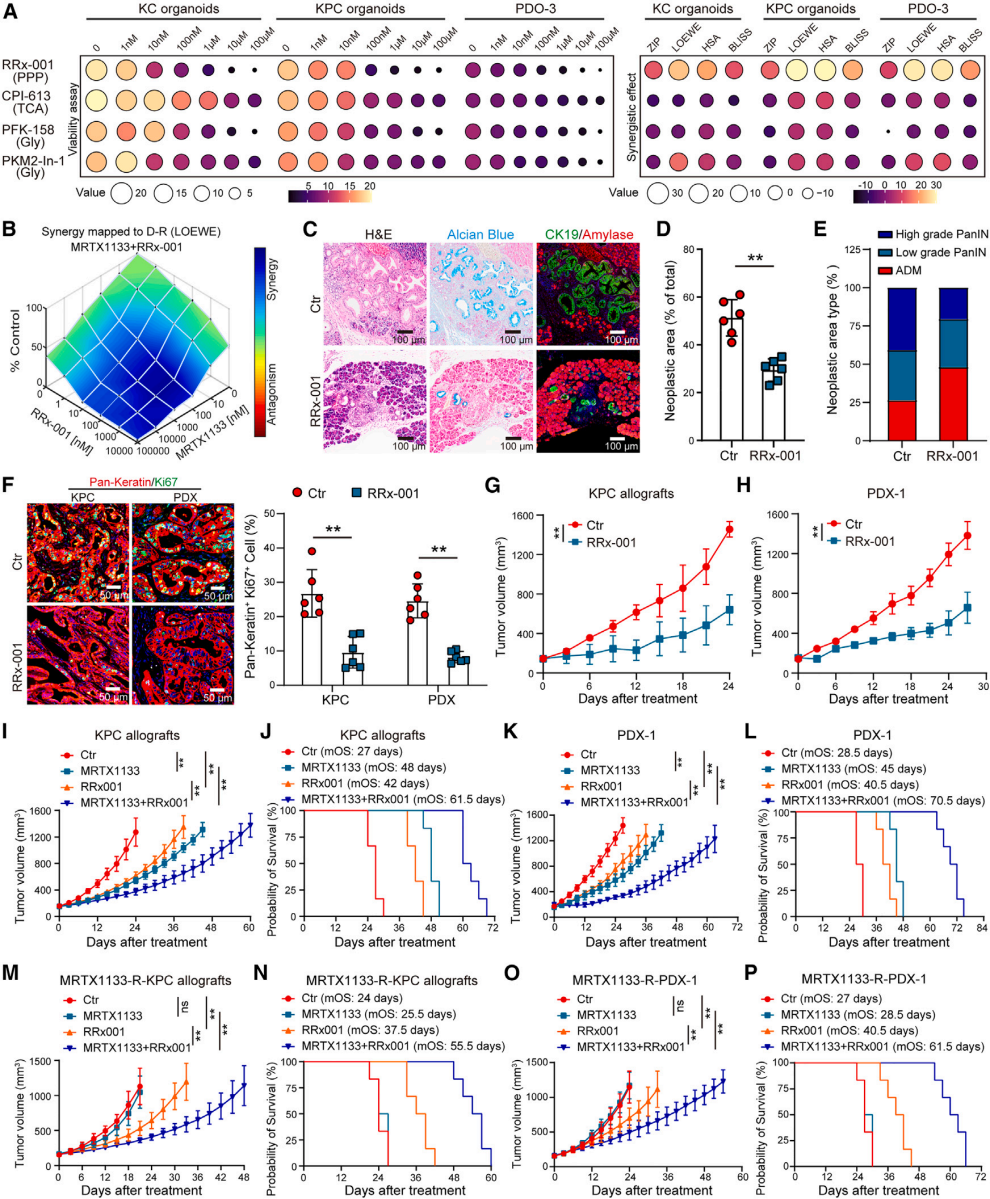
G6PD inhibition reduces malignancy and resistance to MRTX1133 in KRAS^G12D^-mutated PDAC (A) Heatmap illustrating organoid area fold changes and synergy indexes with MRTX1133 following treatment with the indicated inhibitors. Measurements taken 6 days post treatment. Organoid area fold changes and synergy indexes are represented by the indicated values of different colors and sizes. (B) Synergy analysis of RRx-001 and MRTX1133 using the Loewe model in KPC organoids. (C–E) Representative images of pancreatic tissues from KC mice stained with H&E, Alcian blue, and amylase/CK19 with or without RRx-001 treatment (5 mg/kg/day) (C). Quantification of the total (D) and differential-grade (E) area of precancerous lesions in the entire pancreatic tissue section (*n* = 6). (F) Representative images and quantification of pan-keratin and Ki67 staining in PDAC tissues from KPC allografts and PDX-1 models, with and without RRx-001 treatment (5 mg/kg/day) (*n* = 6). (G and H) Tumor growth of KPC allografts (G) and PDX-1 (H) models with or without RRx-001 (*n* = 6). (I–L) Tumor growth and survival analysis of KPC allografts (I and J) and PDX-1 (K and L) models treated with RRx-001 (5 mg/kg/day) and/or MRTX1133 (30 mg/kg/day) (*n* = 6). (M–P) Tumor growth and survival analysis of MRTX1133-resistant KPC allografts (M and N) and PDX-1 (O and P) models treated with RRx-001 and/or MRTX1133. (*n* = 6). Mean ± SD, Student’s t test. ∗∗*p* < 0.01, ns, not significant. See also Figures S4.

The effects of inhibitors targeting the PPP (RRx-001), glycolysis (PFK-158 and PKM2-in-1), and the TCA cycle (CPI-613) on growth rates and their synergistic effects with MRTX1133 were evaluated using organoids derived from KC and KPC (harboring *LSL-Kras*^*G12D/+*^, *LSL-Trp53*^*R172H/+*^, *Pdx1-Cre*) mice and patients with PDAC. Among these inhibitors, RRx-001 significantly reduced organoid growth rates and exhibited a pronounced synergistic effect when combined with MRTX1133 (Figures 2A, 2B, S4A, and S4B). RRx-001 treatment substantially diminished the area and grade of precancerous lesions in the pancreatic tissues of KC mice (Figures 2C–2E). This treatment also led to a significant decrease in tumor growth rate and a reduction in Ki67 expression levels in PDAC tissues from both the KPC allografts and patient-derived xenograft (PDX) models (Figures 2F–2H, S4C, and S4D).

Furthermore, the combination therapy of RRx-001 and MRTX1133 substantially inhibited tumor growth and extended survival in the KPC allografts (median overall survival [mOS]: 61.5 vs. 48 days) and PDX models (mOS: 70.5 vs. 45 days) compared with that using MRTX1133 monotherapy (Figures 2I–2L). We observed that RRx-001 reversed the acquired MRTX1133 resistance developed in KPC allografts and PDX models. The combination of RRx-001 and MRTX1133 demonstrated a significantly longer mOS in both the MRTX1133-resistant KPC allografts (55.5 vs. 25.5 days) and PDX (61.5 vs. 28.5 days) models compared with that in the MRTX1133 monotherapy models (Figures 2M–2P).

### KRAS^G12D^ promotes PPP reprogramming through UBE2T-mediated ubiquitination and degradation of p53

**Figure 3:**
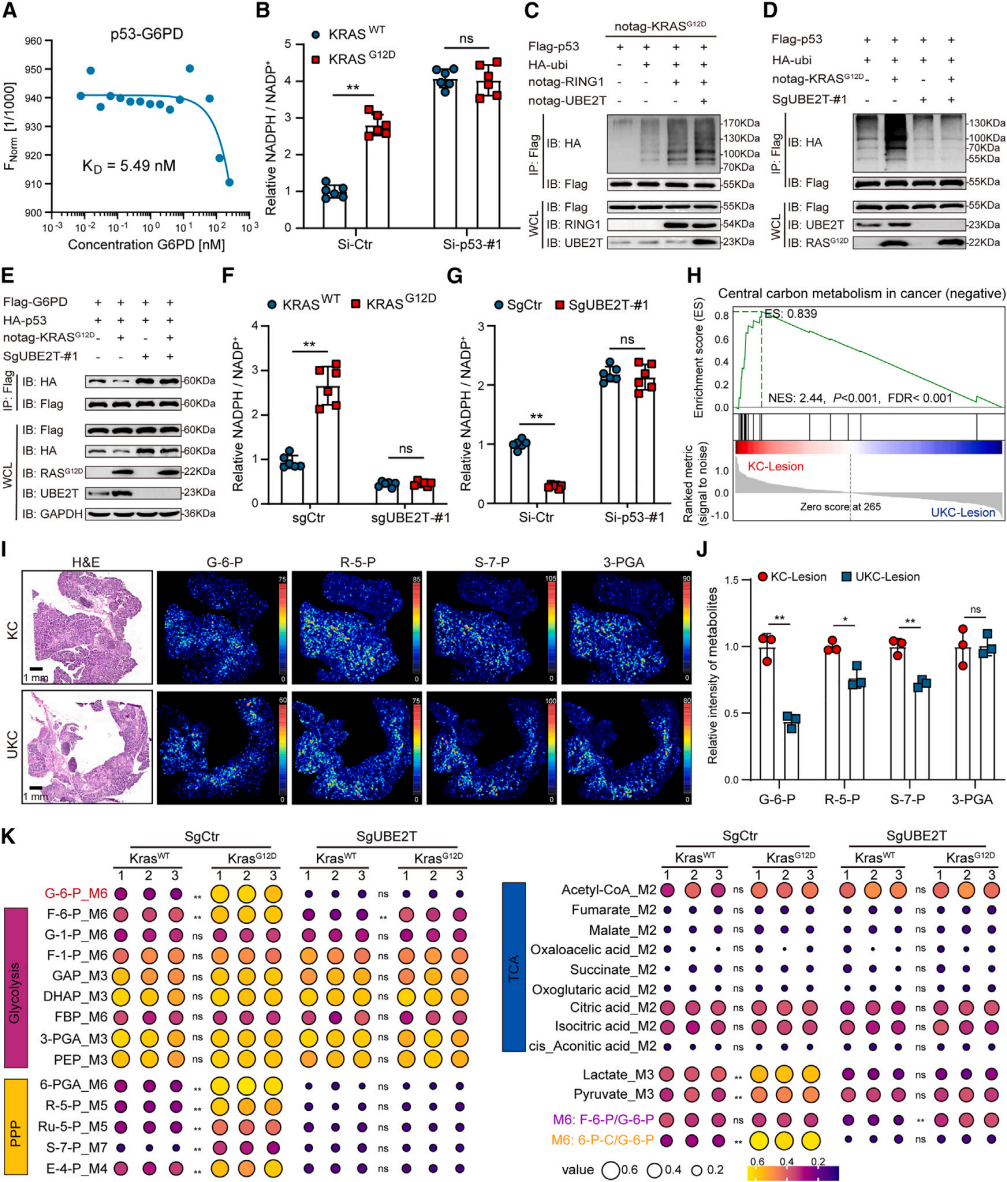
KRAS^G12D^ drives PPP reprogramming through UBE2T-mediated p53 ubiquitination (A) Microscale thermophoresis (MST) curve displaying the interaction between p53 and G6PD. K_D_, the equilibrium dissociation constant. (B) Detection of G6PD enzyme activity using NADPH/NADP^+^ ratio in KRAS^WT^ or KRAS^G12D^ PDO-1 with or without *TP53* knockdown (*n* = 6). (C and D) Ubiquitination assay illustrating the degree of p53 ubiquitination in HEK-293T (C) and BxPC-3 (D) cells expressing the indicated plasmids. (E) Co-immunoprecipitation (coIP) assays reveal the interaction between p53 and G6PD in control (SgCtr) or *UBE2T*-knockout (SgUBE2T) BxPC-3 cells coexpressing the indicated plasmids. (F) G6PD enzyme activity in KRAS^WT^ or KRAS^G12D^ PDO-1 with or without *UBE2T* deletion (*n* = 6). (G) G6PD enzyme activity in SgCtr or SgUBE2T PDO-3 with or without *TP53* knockdown (*n* = 6). (H) GSEA of differential metabolites from lesion tissues of KC or UKC mice based on spatial metabolomics data. (I and J) H&E staining and MSI of G-6-P, R-5-P, S-7-P, and 3-PGA (I), followed by statistical analysis (*n* = 3) (J). (K) Heatmap showing the indicated metabolites level from U-^13^C_6_-labeled metabolic flux analysis in WT and G12D-mutant PDO-1 with or without *UBE2T* knockout (*n* = 3). See Table S1. Mean ± SD, Student’s t test. ∗*p* < 0.05, ∗∗*p* < 0.01, ns, not significant. See also Figures S5.

The glucose flux of PPP is regulated by p53; the p53 protein directly binds to G6PD and inactivates its enzyme activity.^28^^,^^29^ Concordantly, our findings revealed a significant correlation between KRAS^G12D^ and the p53 signaling pathway, which further substantiates the pivotal role of p53 in KRAS^G12D^-driven metabolism regulation (Figure S1B). We demonstrated a direct interaction between p53 and G6PD (Figure 3A), and the knockdown of *TP53* substantially enhances G6PD enzyme activity (Figures 3B and S5A). Notably, the KRAS^G12D^ overexpression in the *TP53*-knockdown PDOs could not enhance G6PD enzyme activity (Figures 3B and S5A).

KRAS^G12D^ leads to the abnormal activation of the proteasome pathway (Figure S1B), which primarily contributes to the loss of p53. We previously reported that UBE2T, a ubiquitin-conjugating enzyme, can interact with the ubiquitin ligase RING1 to facilitate the ubiquitination and subsequent degradation of p53.^30^ This degradation pathway is similarly activated in the context of KRAS^G12D^ mutations (Figure 3C). KRAS^G12D^ enhances p53 ubiquitination, diminishes the interaction between p53 and G6PD, and ultimately increases G6PD enzyme activity (Figures 3D–3F). Conversely, the absence of *UBE2T* significantly impedes p53 ubiquitination, strengthens the p53-G6PD interaction, and reduces G6PD activity (Figures 3D–3F). In *UBE2T*-knockout cell lines, KRAS^G12D^ overexpression did not alter p53 ubiquitination levels, the p53-G6PD interaction, or G6PD activity (Figures 3D–3F and S5B–S5D). Moreover, *TP53* knockdown counteracts the influence of *UBE2T* deletion on G6PD activity (Figures 3G and S5E).

To assess the impact of UBE2T on the KRAS^G12D^-driven PPP, we employed spatial metabolomics to evaluate the metabolic profiles within different regions of KC or UKC mice. We observed a substantial decrease in the levels of central carbon metabolism within the lesion areas of tumors from *Ube2t*-deficient KC mice (Figures 3H and S5F–S5H). Furthermore, the significant downregulation of key metabolites was associated with the PPP pathway (Figures 3I and 3J). Targeted metabolomics on central carbon metabolism in KRAS^G12D^-mutant PDOs with or without the *UBE2T* deletion indicated that *UBE2T* deletion had the most pronounced impact on the PPP (Figure S5I). Furthermore, KRAS^G12D^ overexpression resulted in increased levels metabolites within the PPP pathway. U-^13^C_6_-labeled metabolic flux analysis revealed that the *UBE2T* deletion was also associated with the downregulation of these metabolites in KRAS^G12D^ PDOs (Figure 3K; Table S1). In PDOs with the *UBE2T* deletion, the effect of KRAS^G12D^ on these metabolites was counteracted (Figure 3K; Table S1). These findings highlight the pivotal role of UBE2T in KRAS^G12D^-mediated PPP regulation.

### KRAS^G12D^ establishes positive feedback loops that amplify *UBE2T* transcription through the E2F1/Rb/p53 axis

**Figure 4:**
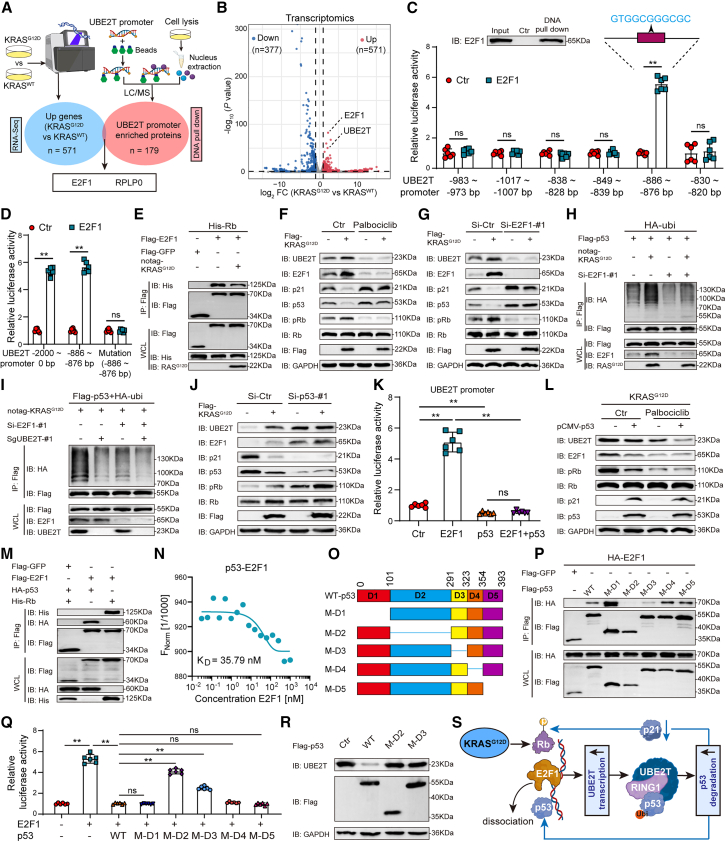
KRAS^G12D^ amplifies *UBE2T* transcription by Rb/E2F1/p53 axis-mediated positive feedback loops (A) Schematic diagram illustrating the identification of *UBE2T* transcription factors. (B) Volcano plot showing DEGs between KRAS^WT^ and KRAS^G12D^ PDO-1. (C) DNA pull-down assay showing the interaction of E2F1 with *UBE2T* promoter (top). Dual-luc assays detecting the transcriptional activity of the indicated *UBE2T* promoter with or without E2F1 overexpression (bottom) (*n* = 6). (D) Dual-luc assays detecting the transcriptional activity of *UBE2T* promoter (full length, −886 to −876 bp, and its mutant version) with or without E2F1 overexpression (*n* = 6). (E) CoIP assays showing the interaction between Rb and E2F1 in BxPC-3 cells. Green fluorescent protein (GFP) as control. (F and G) Immunoblotting (IB) analysis with the indicated antibodies in control or KRAS^G12D^-overexpressed BxPC-3 cells with or without palbociclib treatment (F)/E2F1 knockdown (G). (H and I) Ubiquitination assay showing the degree of p53 ubiquitination using BxPC-3 cells expressing the indicated plasmids. (J) IB analysis with the indicated antibodies in control or KRAS^G12D^-overexpressed BxPC-3 cells with or without *TP53* knockdown. (K) Dual-luc assays detect the transcriptional activities of the *UBE2T* promoter (−886 to −876 bp) with or without E2F1 and/or p53 overexpression (*n* = 6). (L) IB analysis with the indicated antibodies in KRAS^G12D^-overexpressed BxPC-3 cells with or without p53 overexpression and/or palbociclib treatment. (M) CoIP assays assess the interaction of E2F1 with Rb or p53 in BxPC-3 cells expressing the indicated plasmids. (N) MST curve showing the interaction between p53 and E2F1. (O) Schematic diagram of the generation of deletion-mutation p53. (P) CoIP assays detect the interaction between E2F1 and p53 mutants in HEK-293T cells expressing the indicated plasmids. (Q) Dual-luc assays detect the transcriptional activities of the *UBE2T* promoter (−886 ∼ −876 bp) with or without E2F1 and/or p53-mutant overexpression (*n* = 6). (R) IB analysis with the indicated antibodies in KRAS^G12D^-overexpressed BxPC-3 cells expressing the indicated p53-mutant plasmids. (S) Schematic diagram of regulatory mechanism. Mean ± SD, Student’s t test. ∗∗*p* < 0.01, ns, not significant. See also Figures S6.

We observed that KRAS^G12D^ promotes the transcription of *UBE2T* (Figures S6A and S6B). By identifying the truncating mutations in the promoter region of *UBE2T* and conducting dual-luciferase reporter gene (dual-luc) assays, we observed that KRAS^G12D^ specifically activated the transcription of the *UBE2T* promoter within the −1,200 to −800 bp region (Figure S6C). Consequently, we performed DNA pull-down assays in combination with the upregulated gene from the transcriptomics of KRAS^WT^ or KRAS^G12D^ PDOs to identify transcription factors for *UBE2T* (Figures 4A and 4B). We observed that ribosomal protein large P0 (RPLP0), a ribosomal protein, did not bind to the *UBE2T* promoter (Figure S6D). However, E2F transcription factor 1 (E2F1) interacted with the *UBE2T* promoter (Figure 4C). A detailed promoter sequence was predicted within the −1,200 to −800 bp region using the JASPAR database, and we observed that E2F1 promoted *UBE2T* transcription within the −886 to −876 bp region (Figures 4C and 4D). The mRNA expression of *UBE2T* is positively correlated with E2F1 (Figure S6E).

Retinoblastoma (Rb) is a key factor in regulating the transcriptional activity of E2F1. The binding of Rb to E2F1 restricts the transcriptional activity of E2F1. Conversely, Rb phosphorylation releases E2F1, enhancing both its expression and transcriptional activity.^31^^,^^32^ Moreover, the extracellular regulated protein kinases (ERK) downstream of KRAS^G12D^ is an essential activator required for Rb phosphorylation.^33^ Therefore, we hypothesize that KRAS^G12D^ likely promotes Rb phosphorylation through ERK, and consequently, enhances E2F1-mediated transcription of *UBE2T* and facilitates p53 degradation. Our data confirmed that KRAS^G12D^ promotes Rb phosphorylation depended on ERK phosphorylation, and its overexpression facilitates the dissociation between Rb and E2F1, thereby upregulating E2F1 and UBE2T, and then downregulating p53 (Figures 4E, 4F, and S6F). Additionally, inhibition of Rb or ERK phosphorylation and knockdown of Rb diminish these regulatory impacts with or without KRAS^G12D^ overexpression (Figures 4F, S6F, and S6G). Knockdown of *E2F1* reduced UBE2T expression, decreased p53 degradation, and diminished G6PD enzyme activity, suggesting that the regulatory effects of KRAS^G12D^ on UBE2T/p53/G6PD depend on E2F1 (Figures 4G, 4H, and S6H–S6M). The absence of *UBE2T* mitigates p53 ubiquitination induced by KRAS^G12D^/E2F1 and dampens the effect of E2F1 on this process (Figures 4I and S6N).

We demonstrated that *TP53* knockdown enhances Rb phosphorylation, which in turn increases E2F1 and UBE2T expression, with KRAS^G12D^ overexpression in such cells failing to further amplify E2F1 and UBE2T levels (Figures 4J and S6O–S6Q). Moreover, p53 overexpression inhibited the transcription of *UBE2T* by E2F1 (Figures 4K and 4L). p53 inhibits the phosphorylation of Rb through p21,^30^ which is consistent with our findings. These data suggest that p53 participates in feedback regulation, controlling UBE2T transcription by E2F1 through the inhibition of Rb phosphorylation via p21. Notably, with palbociclib treatment, p53 overexpression did not inhibit Rb phosphorylation or E2F1 expression but still downregulated UBE2T expression (Figures 4L and S6R), indicating that p53 also regulates UBE2T transcription through alternative pathways. E2F1 and p53 exhibit extensive crosstalk, with p53 interacting with E2F1 to inhibit its transcriptional activity.^34^ Our data suggested that E2F1 directly binds with p53 and Rb to form a complex (Figures 4M, 4N, and S6S). To investigate the regulatory effect of the interaction between p53 and E2F1 on the transcription of *UBE2T*, five deletion mutants of p53 were constructed based on its functional domains (Figure 4O). Immunoprecipitation assay revealed that p53 mutants lacking the second and third domains exhibited weaker interactions with E2F1 (Figure 4P). Overexpression of these two p53 mutants also failed to effectively inhibit the transcription of *UBE2T* by E2F1 (Figures 4Q, 4R, and S6T).

Collectively, these results suggest that UBE2T-mediated degradation of p53 promotes E2F1-mediated *UBE2T* transcription through a positive feedback mechanism involving the regulation of Rb phosphorylation and the interaction between Rb and E2F1 (Figure 4S).

### UBE2T promotes malignant progression and impairs MRTX1133 efficacy in KRAS^G12D^-mutant PDAC

UBE2T is significantly overexpressed in PDAC and was identified as a pivotal element in feedback regulation, highlighting its therapeutic target potential in KRAS^G12D^-driven PDAC. The UBE2T expression level was significantly higher in the KRAS^G12D^-mutated PDAC samples compared with those with the KRAS^WT^ genotype (Figure S7A). We collected 160 PDAC tissue samples and found that the KRAS^G12D^ and UBE2T protein levels are positively correlated (Figure S7B). Among patients with high levels of RAS^G12D^ expression (*n* = 110), those with high levels of UBE2T expression had a worse survival prognosis compared with patients with low UBE2T expression (Figure S7C). Cox proportional hazards analysis revealed that UBE2T could serve as an independent prognostic predictor for patients with high RAS^G12D^ expression (Figures S7D and S7E).

**Figure 5:**
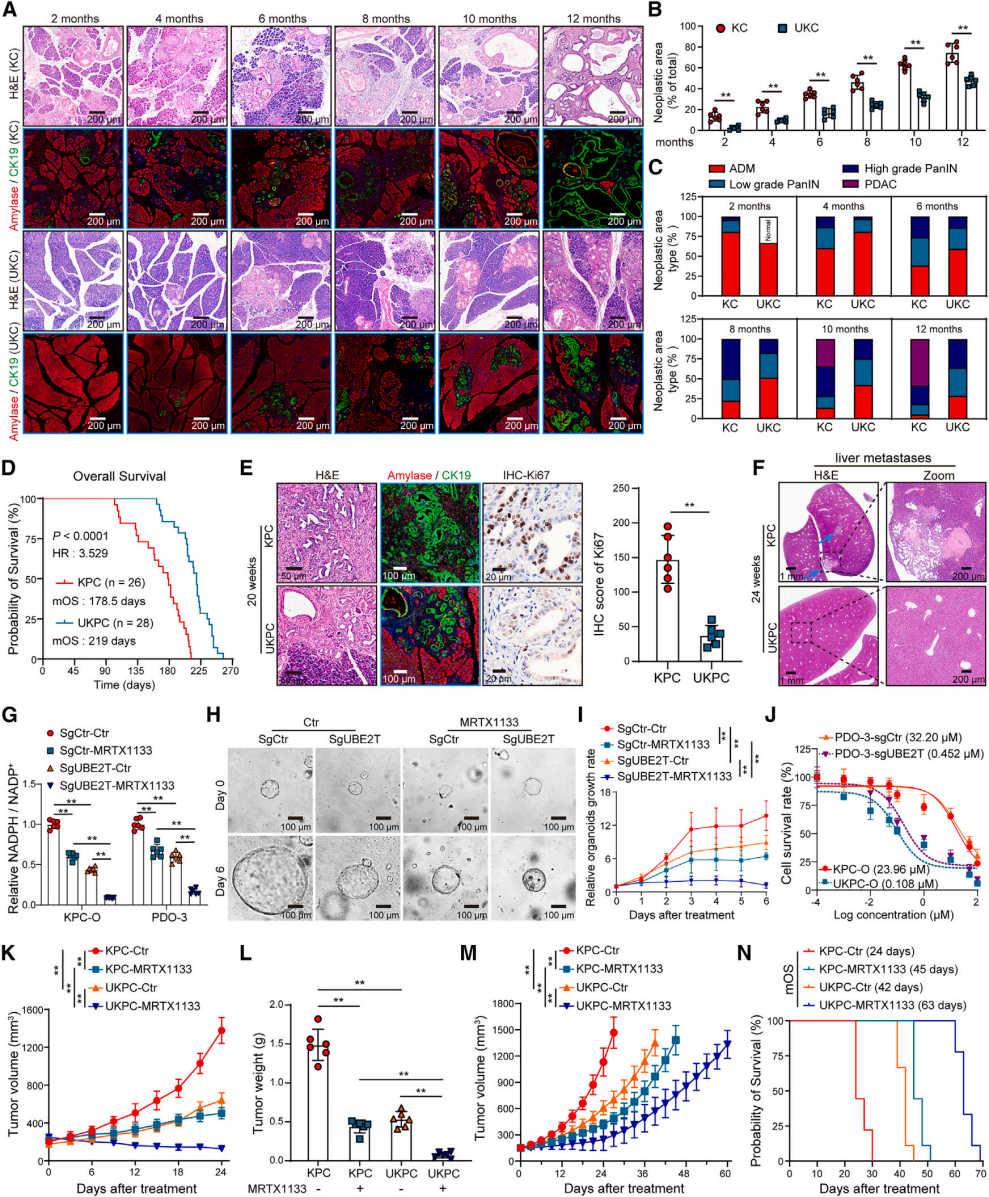
Genetic ablation of *UBE2T* inhibits malignant progression and potentiates MRTX1133 efficacy in KRAS^G12D^-mutant PDAC (A–C) Pancreatic tissues from KC and UKC mice aged 2, 4, 6, 8, 10, and 12 months, stained with H&E and amylase/CK19 (A). Quantification of the total (B) and differential-grade (C) area of precancerous lesions in the entire pancreatic tissue section (*n* = 6). (D) Kaplan-Meier survival curves with log-rank test comparing overall survival between KPC and UKPC mice. (E) H&E, amylase/CK19, and Ki67 staining of PDAC tissues from 20-week-old KPC and UKPC mice (left). Quantification of Ki67 level (right) (*n* = 6). (F) H&E staining of liver tissues from 24-week-old KPC and UKPC mice. (G) G6PD enzyme activity measured by NADPH/NADP^+^ ratio in SgCtr or SgUBE2T PDO-3 and KPC or UKPC organoids with or without MRTX1133 treatment (10 μM, *n* = 6). (H and I) Representative images (H) and quantification (I) of the response of SgCtr or SgUBE2T PDO-3 response to MRTX1133 (10 μM, *n* = 6). (J) Sensitivity of SgCtr or SgUBE2T PDO-3 and KPC or UKPC organoids to MRTX1133 (*n* = 6). (K and L) Tumor growth (K) and tumor weight (L) of KPC or UKPC allografts models with or without MRTX1133 treatment (30 mg/kg/day) (*n* = 6). (M and N) Tumor growth (M) and survival analysis (N) of KPC or UKPC allografts treated with or without MRTX1133 (*n* = 9). Mean ± SD, Student’s t test. ∗∗*p* < 0.01. See also Figures S7 and S8.

We observed that *Ube2t* ablation significantly reduced the area and grade of precancerous lesions in KC mice and eventually delayed the development of PDAC (Figures 5A–5C and S8A). The absence of *Ube2t* also decreased G6PD enzyme activity in KC organoids (Figure S8B). Further investigation using KPC models with or without *Ube2t* deletion (UKPC mice) demonstrated that the *Ube2t* knockout prolonged the overall survival (OS) of the KPC mice (mOS: 219 vs. 178.5 days) (Figure 5D). Considering the typical development of invasive ductal carcinoma by 20 weeks and liver metastasis by 24 weeks in KPC mice,^35^ histopathologic examinations were performed at these time points. We observed that UKPC mice exhibited a less malignant disease course and reduced metastasis rates (Figures 5E, 5F, and S8C). These findings were further validated in allograft models derived from 24-week-old KPC and UKPC mice, with UKPC-derived tumors showing diminished growth and reduced proliferation markers (Figures S8D–S8G). The invasive capabilities of UKPC organoids were also significantly weaker compared with those of KPC organoids (Figure S8H).

Combining the *UBE2T* knockout with MRTX1133 treatment substantially altered key regulatory proteins and decreased E2F1 and phosphorylated Rb levels while upregulating p53 and p21 (Figures S8I and S8J). Both the *UBE2T* knockout and MRTX1133 treatment individually downregulated G6PD activity, and when combined, a more pronounced effect on PDO growth rates was observed (Figures 5G–5I). Cell viability assays revealed that *UBE2T* deficiency significantly reduced the IC50 of MRTX1133 for PDOs and KPC organoids, which consequently enhanced treatment sensitivity (Figure 5J). *In vivo*, UKPC mice treated with MRTX1133 saw reduced tumor growth, as well as extended survival times (mOS: 63 vs. 45 days), compared with that of KPC mice (Figures 5K–5N, S8K, and S8L). In UKPC mice, tumor growth is slower, which may result in more pronounced drug effects compared to KPC mice. Additionally, UBE2T overexpression reduces MRTX1133 sensitivity both *in vitro* and *in vivo* (Figures S8M–S8P).

### Pharmacological inhibition of UBE2T regulates PPP reprogramming to inhibit malignant progression and overcome MRTX1133 resistance

**Figure 6:**
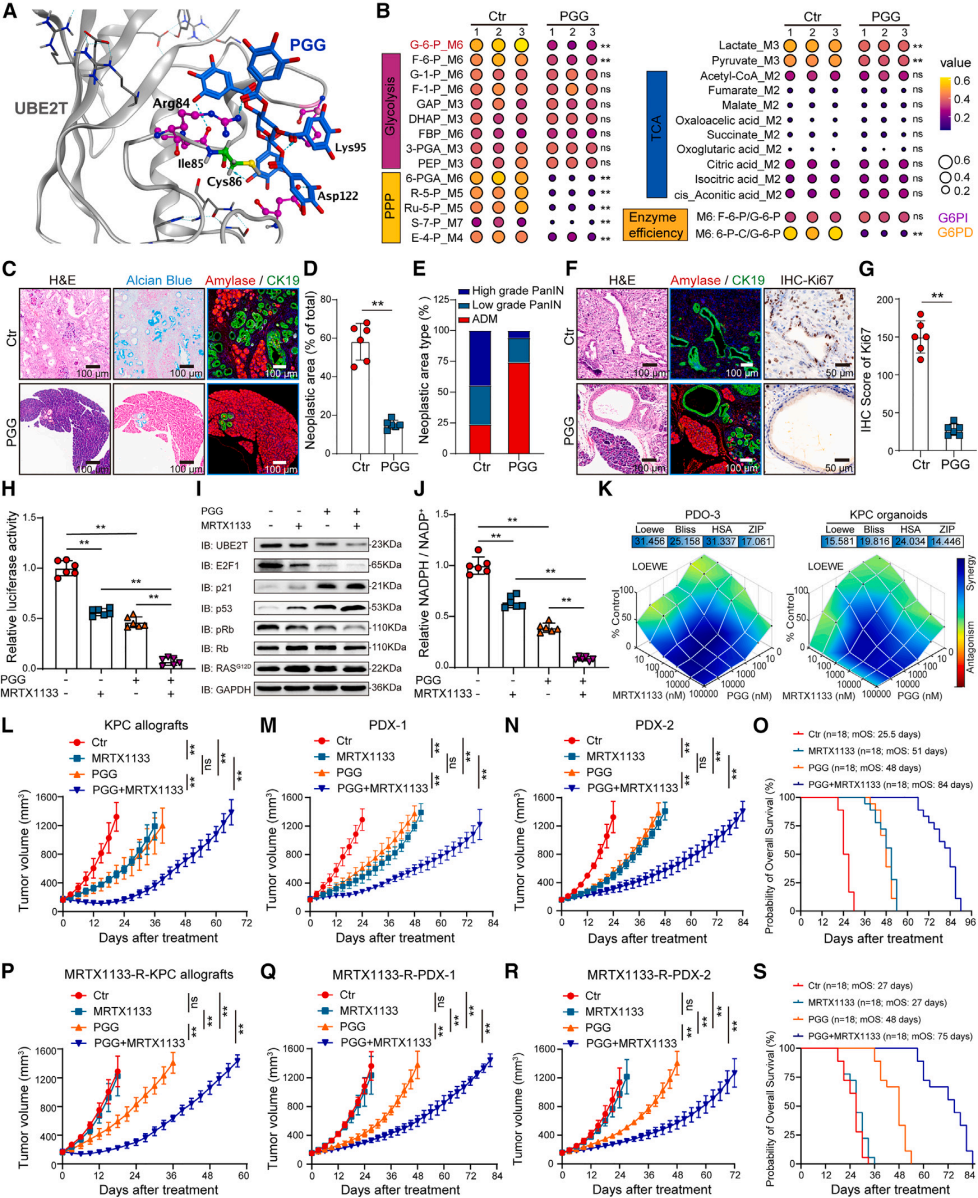
UBE2T inhibitor PGG suppresses malignant progression and MRTX1133 resistance by regulating PPP reprogramming (A) Computational model and interactions of PGG and UBE2T. (B) Heatmap displaying the indicated metabolites level from U-^13^C_6_-labeled metabolic flux analysis in PDO-3 with or without PGG treatment (10 μM) (*n* = 3). See Table S1. (C–E) Representative images of pancreatic tissues stained with H&E, Alcian blue, and amylase/CK19 with or without PGG treatment (40 mg/kg/day) (C). Quantification of the total (D) and differential-grade (E) area of precancerous lesions in the entire pancreatic tissue section (*n* = 6). (F) Representative images of PDAC tissues stained with H&E, amylase/CK19, and Ki67 in 20-week-old KPC mice with or without PGG treatment. (G) Quantification of the Ki67 level (*n* = 6). (H) Dual-luc assays detect the UBE2T promoter activity (positions −886 to −876 bp) with or without PGG (10 μM) and/or MRTX1133 (10 μM) treatment (*n* = 6). (I) IB analysis with the indicated antibodies in PDO-3 with or without PGG (10 μM) and/or MRTX1133 (10 μM) treatment. (J) G6PD enzyme activity in PDO-3 with or without PGG (10 μM) and/or MRTX1133 (10 μM) treatment (*n* = 6). (K) Synergy analysis of PGG and MRTX1133 in PDO-3 and KPC organoids using the Loewe, Bliss, HSA, and ZIP model. (L–N) Tumor growth of KPC allografts (L) and PDX-1 (M) and PDX-2 (N) models treated with PGG (40 mg/kg/day) and/or MRTX1133 (30 mg/kg/day) (*n* = 6). (O) Overall survival analysis of KPC allografts and PDX-1 and PDX-2 models treated with PGG and/or MRTX1133. (P–R) Tumor growth of MRTX1133-resistant KPC allografts (P) and PDX-1 (Q) and PDX-2 (R) models treated with PGG and/or MRTX1133 (*n* = 6). (S) Overall survival analysis of MRTX1133-resistant KPC allografts and PDX-1 and PDX-2 models treated with PGG and/or MRTX1133. Mean ± SD, Student’s t test. ∗∗*p* < 0.01, ns, not significant. See also Figures S9–S11.

We previously identified pentagalloylglucose (PGG) as a highly selective inhibitor of UBE2T (Figures 6A and S9A). In this study, U-^13^C_6_-labeled metabolic flux analysis revealed that PGG significantly reduced glucose-6-phosphate level and decreased glucose flux through the G6PD-mediated PPP, highlighting the potent role of PGG in regulating central carbon metabolism (Figure 6B; Table S1). We also observed that PGG effectively inhibited the growth of organoids derived from patients and KC and KPC mice (Figures S9B–S9E). PGG treatment also significantly reduced the area and severity of precancerous lesions in KC mice (Figures 6C–6E) and decreased the malignancy of PDAC in KPC mice (Figures 6F and 6G). Furthermore, the combination of PGG and MRTX1133 significantly inhibited the signaling pathway of the Rb-E2F1-UBE2T-p53 axis and reduced G6PD enzyme activity compared with that using MRTX1133 monotherapy (Figures 6H–6J and S9F–S9H).

The Loewe, Bliss, highest single agent (HSA), and zero interaction potency (ZIP) models for calculating drug synergy revealed synergistic effects between PGG and MRTX1133 (Figures 6K, S9I, and S9J). *In vivo*, the combination of PGG and MRTX1133 significantly inhibited tumor growth in the KPC allografts, as well as those models with acquired MRTX1133 resistance (Figure S10). We observed that the combination of MRTX1133 and PGG delayed tumor growth and extended OS of the KPC allografts (mOS: 70.5 vs. 42 days) and PDX-1 (mOS: 85.5 vs. 54 days), PDX-2 (mOS: 87 vs. 51 days), and all models (mOS: 84 vs. 51 days) compared with that using MRTX1133 monotherapy (Figures 6L–6O and S11A–S11F). Furthermore, the combination of PGG and MRTX1133 extended the OS of MRTX1133-resistant KPC allografts (mOS: 60 vs. 21 days) and PDX-1 (mOS: 79.5 vs. 30 days), PDX-2 (mOS: 79.5 vs. 30 days), and all models (mOS: 75 vs. 27 days) (Figures 6P–6S and S11G–S11L).

### MFP nano-delivery system achieves durable response and long-term survival in PDAC with KRAS^G12D^ mutation

**Figure 7:**
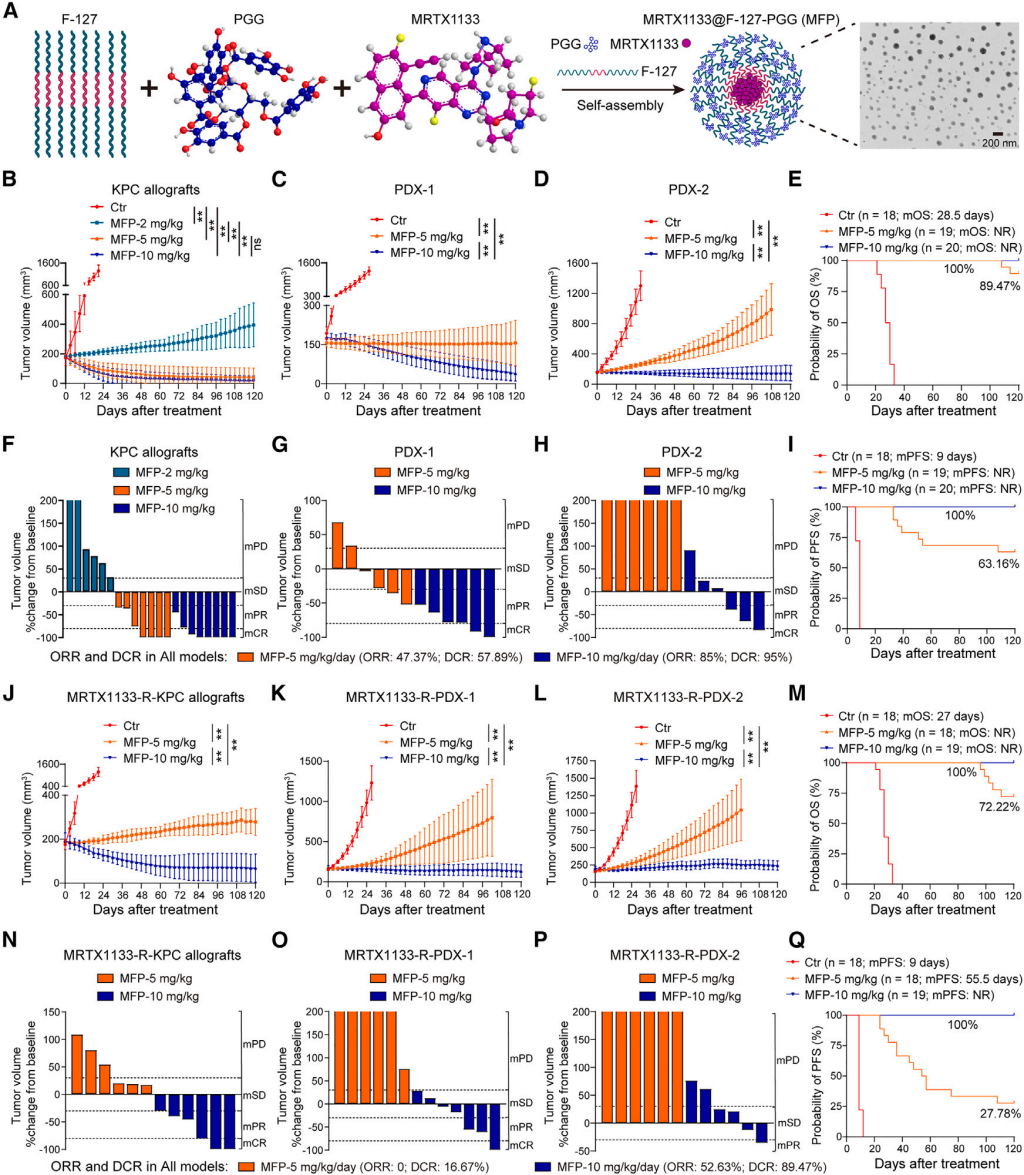
MFP shrinks tumor volume and sustains long-term survival in PDAC with KRAS^G12D^ mutation (A) Schematic diagram of MFP nano-delivery system construction. (B–D) Tumor growth of KPC allografts (B) and PDX-1 (C) and PDX-2 (D) models treated with MFP (*n* ≥ 6). (E) Overall survival of KPC allografts and PDX-1 and PDX-2 models treated with MFP. NR, not reached. (F–H) Fold changes of tumor volume in KPC allografts (F) and PDX-1 (G) and PDX-2 (H) models treated with MFP at 120 days (*n* ≥ 6). mPD, progressive disease; mSD, stable disease; mPR, partial response; mCR, complete response. (I) PFS of KPC allografts and PDX-1 and PDX-2 models treated with MFP. (J–L) Tumor growth of MRTX1133-resistant KPC allografts (J) and PDX-1 (K) and PDX-2 (L) models treated with MFP (*n* ≥ 6). (M) Overall survival of MRTX1133-resistant KPC allografts and PDX-1 and PDX-2 models treated with MFP. (N–P) Fold changes of tumor volume in MRTX1133-resistant KPC allografts (N) and PDX-1 (O) and PDX-2 (P) models treated with MFP at 120 days (*n* ≥ 6). (Q) PFS of MRTX1133-resistant KPC allografts and PDX-1 and PDX-2 models treated with MFP. Mean ± SD, Student’s t test. ∗∗*p* < 0.01; ns, not significant. See also Figures S12–S14.

The limited bioavailability of MRTX1133 and PGG, owing to their poor solubility and the requirement for high therapeutic doses, restricts their clinical efficacy.^6^^,^^36^ PGG, a polyphenolic compound, exhibits strong intermolecular interactions with the amphiphilic polymer pluronic F-127 and facilitates self-assembly into a nanomedicine delivery system. F-127 is recognized for its non-toxicity, biocompatibility, and bioabsorbability, garnering Food and Drug Administration (FDA) approval as a pharmaceutical excipient. Utilizing F-127, we developed a nano-delivery system capable of co-delivering PGG and MRTX1133 (MRTX1133@F-127-PGG, MFP). Transmission electron microscopy revealed that the MFP system is well dispersed with a uniform size distribution (Figure 7A). Its hydrodynamic diameter in aqueous solutions is approximately 100 nm and exhibited enhanced stability in such environments (Figures S12A and S12B). Spectroscopic analyses via UV-visible absorption, Fourier transform infrared, and nuclear magnetic resonance confirmed the presence of characteristic absorption peaks for both PGG and MRTX1133, which indicated strong F-127 and PGG interactions (Figures S12C–S12E). The MFP system exhibits a controlled release behavior, with MRTX1133 release rates under normal physiological conditions (pH 7.4) below 20%. However, in the acidic microenvironment typical of tumors (pH 5.0), the release rate reached 75% (Figure S12F). Drug content analysis via nuclear magnetic resonance spectroscopy indicated that PGG and MRTX1133 constitute 23.66% and 20.50% of the system, respectively (Figure S12G).

To examine the therapeutic effects of the MFP nano-delivery system on KRAS^G12D^-mutated PDAC, we used the loading of MRTX1133 as a reference point. The treatment efficacy was then evaluated across MRTX1133 dosages of 2, 5, and 10 mg/kg/day within the MFP system. We observed that dosages of 5 and 10 mg/kg/day helped achieve tumor shrinkage and facilitated long-term survival of the KPC allografts (Figures 7B, S13A, and S13D). Therefore, these two therapeutic doses (5 and 10 mg/kg/day) were selected for subsequent experiments. Administration of 10 mg/kg/day MFP resulted in substantial tumor regression and extended OS in both PDX-1 and PDX-2 models. A lower dose of 5 mg/kg/day effectively suppressed tumor growth (Figures 7C, 7D, and S13B–S13F). Comprehensive survival analysis demonstrated high OS rates in mice treated with MFP (10 mg/kg/day: 100%; 5 mg/kg/day: 89.47%) (Figure 7E). Modified response evaluation criteria were used to assess the solid tumor (RECIST) responses to MFP. Tumor responses were classified as modified progressive disease, modified stable disease, modified partial response, and modified complete response.^37^ The 10 mg/kg/day MFP exhibited the highest efficacy, with an overall response rate (ORR) of 85% and a disease control rate (DCR) of 95% (Figures 7F–7H). The 10 mg/kg/day MFP group achieved a 100% progression-free survival (PFS) rate within 120 days, while the 5 mg/kg/day group reached 63.16% (Figures 7I and S13G–S13I). Furthermore, we found that the KPC model exhibited a stronger therapeutic response to MFP compared to the PDX models, potentially due to enhanced CD8^+^ T cell infiltration mediated by MRTX1133. Our results also demonstrated that MFP enhances CD8^+^ T cell infiltration (Figures S13J and S13K).

In models with acquired MRTX1133 resistance, the 10 mg/kg/day MFP dosage continued to induce tumor regression and helped achieve 100% OS, whereas 72.22% OS could be achieved with the 5 mg/kg/day dosage (Figures 7J–7M and S14A–S14F). Most mice also achieved a sustained response with the 10 mg/kg/day MFP treatment; the observed ORR was 52.63%, and the DCR was 89.47% (Figures 7N–7P). At this dosage, the PFS rate remained at 100% within 120 days, whereas it reached 27.78% at 5 mg/kg/day (Figures 7Q and S14G–S14I). Additionally, we evaluated the acute toxicity of MFP. The results demonstrated that administration of MFP at a dose of 10 mg/kg/day did not significantly affect body weight and food or water intake, nor did it result in any discernible organ toxicity (Figures S14J–S14M), indicating that MFP has a good safety profile.

## Discussion

The KRAS^G12D^ mutation is a key factor leading to high levels of malignancy and treatment resistance for PDAC.^38^^,^^39^ MRTX1133 has shown promising therapeutic potential in PDAC but still faces challenges related to resistance, with its regulatory mechanisms remaining unclear. Here, we demonstrate that KRAS^G12D^ drives a PPP-dominant central carbon metabolism pattern, contributing to PDAC progression and resistance to MRTX1133. Mechanistically, KRAS^G12D^ establishes positive feedback loops promoting UBE2T transcription via the Rb/E2F1/UBE2T/p53 axis, leading to p53 degradation and PPP reprogramming. Notably, we developed a nano-delivery system incorporating F-127, UBE2T inhibitors, and MRTX1133 (MFP), which enhanced the efficacy of both PGG and MRTX1133, showing substantial effectiveness in treating KRAS^G12D^-mutant PDAC.

KRAS mutations enhance the uptake of glucose and its direct intermediates into multiple branching pathways, which supports the malignant behavior of cancer cells.^17^^,^^26^ Our ^13^C-labeled metabolic flux analysis revealed the preference of KRAS^G12D^ for the PPP branch in PDAC. The abnormally activated PPP promotes ribose biogenesis and increases resistance to oxidative stress,^40^ which are factors directly associated with the high malignancy of KRAS^G12D^-driven PPP-mediated PDAC. PPP is reportedly pivotal in the progression and recurrence of cancers with KRAS mutations.^20^^,^^41^^,^^42^^,^^43^ Recent reports indicate that MRTX1133 inhibits mammalian target of rapamycin (mTOR) signaling, which has been implicated in acquired resistance to MRTX1133.^44^^,^^45^ Both mTORC1 and mTORC2 enhance glucose flux into the PPP, thereby promoting nucleotide synthesis and cell proliferation.^46^^,^^47^^,^^48^ These findings emphasize the critical role of PPP reprogramming in MRTX1133 resistance.

Our findings revealed that KRAS^G12D^ enhances the transcription of *UBE2T* by E2F1 via Rb phosphorylation, which in turn promotes the degradation of p53 and upregulates G6PD enzyme activity. The absence of p53 further promotes Rb phosphorylation, releases its interaction with E2F1, and provides positive feedback that enhances *UBE2T* transcription. Extensive interactions occur between p53 and the E2F1/Rb complex; notably, E2F1 regulates the stability of the p53 protein through various pathways, whereas p53 modulates the transcriptional activity of E2F1 through feedback mechanisms.^34^^,^^49^^,^^50^ Furthermore, feedback signaling mechanisms within KRAS-associated vertical pathways are pivotal drivers of intrinsic and acquired resistance to KRAS inhibitors.^51^^,^^52^^,^^53^^,^^54^^,^^55^^,^^56^ The reversible and non-covalent binding of MRTX1133 to the KRAS^G12D^ allosteric pocket further increases the possibility of resistance due to downstream feedback mechanisms.^6^ In this study, we identified downstream metabolic reprogramming feedback regulation mechanisms that mediate MRTX1133 resistance. UBE2T serves as the central hub within this feedback loop and is significantly overexpressed in KRAS^G12D^-mutated PDAC. Previous studies have reported the oncogenic role of UBE2T across multiple cancer types.^30^^,^^57^^,^^58^^,^^59^ Here, comprehensive analyses on KRAS^G12D^-mutated PDAC models with *UBE2T* deletion, covering cancer initiation, progression, metastasis, and treatment resistance, have confirmed the oncogenic role of UBE2T. These insights substantiate the potential of targeting UBE2T as a therapeutic strategy for PDAC with the KRAS^G12D^ mutation.

Despite breakthroughs with KRAS^G12D^ inhibitors such as MRTX1133, developing effective combination therapies to enhance efficacy and combat resistance remains a crucial and ongoing task.^6^^,^^60^^,^^61^ In this study, we implemented a combination strategy that simultaneously targets KRAS^G12D^ and UBE2T to treat KRAS^G12D^-mutated PDAC. We previously identified PGG as a highly selective inhibitor of UBE2T with potent antitumor effects in several cancers.^30^^,^^62^^,^^63^^,^^64^ *In vitro*, PGG demonstrated strong synergy with MRTX1133, but this effect was not as pronounced *in vivo*, likely due to the low bioavailability of PGG and MRTX1133. F-127, an FDA-approved pharmaceutical excipient, can help address the low bioavailability issues associated with both MRTX1133 and PGG and aid in improving therapeutic outcomes.^65^ Furthermore, the rich polyphenolic structures of PGG form strong interactions with F-127, which facilitate its dual role as both drug and carrier. Leveraging this unique advantage, we developed a co-delivery nanosystem (MFP) based on F-127, PGG, and MRTX1133. This system exhibits potent antitumor effects at dosages significantly lower than those used in combination treatment strategies involving PGG and MRTX1133. Using this system, we could achieve significant tumor shrinkage and even elimination. Additionally, MFP demonstrates superior efficacy in immunocompetent mice compared with that in PDX models, which may be attributed to the activation of antitumor immunity by KRAS^G12D^ inhibitors.^7^^,^^8^^,^^9^^,^^66^^,^^67^^,^^68^ Therefore, further research is warranted to identify effective MFP combinations with immune checkpoint inhibitors and develop novel therapeutic strategies for PDAC with the KRAS^G12D^ mutation.

In conclusion, our findings elucidate the mechanism by which KRAS^G12D^-driven PPP reprogramming promotes malignant progression and MRTX1133 resistance in PDAC. From a clinical perspective, we propose a therapeutic strategy that involves the UBE2T inhibitor in combination with MRTX1133 treatment. This approach is further improved by a unique nano-delivery system, which has demonstrated potent efficacy in inducing tumor shrinkage and sustaining a durable response. These findings pave the way for novel therapeutic interventions against this challenging disease.

### Limitations of the study

Despite the promising therapeutic effects demonstrated by our MFP nano-delivery system, several limitations warrant consideration. Firstly, our evaluation was conducted using a limited range of PDAC models. Given the high heterogeneity of PDAC, further validation across a more diverse array of models derived from a broader patient population is essential to confirm the generalizability of our findings. Secondly, comprehensive toxicological and pharmacokinetic assessments were not performed in this study. These analyses are crucial to determine the safety profile and clinical feasibility of MFP as a treatment strategy for KRAS^G12D^-mutated PDAC. Additionally, considering the enhanced efficacy of MFP in immunocompetent models and the impact of KRAS^G12D^ on tumor immunity, further investigation into the role of targeting UBE2T in modulating antitumor immune responses is required. These investigations should be complemented by evaluations in large-scale preclinical models to assess the feasibility of combining MFP with immunotherapies as potentially curative approaches for PDAC.

## Resource availability

### Lead contact

Further information and requests for resources and reagents should be directed to and will be fulfilled by the lead contact, Zuoyi Jiao (jiaozy@lzu.edu.cn).

### Materials availability

All unique reagents generated in this study are available from the lead contact without restriction.

### Data and code availability

• Raw data of the RNA-seq have been deposited in the NCBI Sequence Read Archive database under the accession ID PRJNA1201558. Metabolomic data have been deposited at METASPACE annotation platform (https://metaspace2020.eu/project/jiao-2024). Raw data of metabolic flux analysis are available in Table S1. Original western blot images are available at Mendeley Data: https://doi.org/10.17632/z6s7vb8d77.1.
• This paper does not report original code.
• Any additional information required to analyze the data reported in this paper is available from the lead contact upon request.

## Acknowledgments

We extend our appreciation to all colleagues from the Cuiying Biomedical Research Center. Our acknowledgments go to BIOTREE Biotechnology (Shanghai, China), Metabo-Profile (Shanghai, China), and Oebiotech (Shanghai, China) for their professional handling of the transcriptomics and metabolomics analyses. We also thank TissueGnostics (Vienna, Austria) for providing whole-slide bright-field and fluorescence imaging. This work was supported by National Natural Science Foundation for young students basic research project of China (823B2073), 10.13039/501100004775Natural Science Foundation of Gansu Province (24JRRA381), 10.13039/501100001809National Natural Science Foundation of China (8236100425), Major Project Granted from Gansu Provincial Science and Technology Department (22ZD6FA021-4), 10.13039/501100012226Fundamental Research Funds for the Central Universities (lzujbky-2022-ey04), and Lanzhou science and technology project (2024-1-29).

## Author contributions

Conceptualization, X.J., T.W., and Z.J.; software, B.Z., Haonan Sun, M.L., and Z.L.; validation, T.W., B.Z., H.Z., and Y.D.; formal analysis, Y.M., Z.L., Y.W., and K.W.; investigation, X.J., T.W., B.Z., H.Z., Y.D., X.G., Q.H., and W.L.; resources, X.J., Z.J., H.Z., and Z.Y.; data curation, B.L., Y.D., L.Q., W.S., L.X., and L.S.; writing – original draft, X.J., T.W., and Z.J.; writing – review and editing, Z.J., C.Z., J.Y., Hui Sun, and J.G.; visualization, X.J., B.Z., Haonan Sun, and Y.D.; supervision, H.Z., Z.Y., and Z.J.; project administration, Z.J. and Y.D.

## Declaration of interests

The authors declare no competing interests.

## STAR★Methods

### Key resources table

**Table:** 

REAGENT or RESOURCE	SOURCE	IDENTIFIER
**Antibodies**		

Mouse anti-FLAG Monoclonal antibody	Sigma	Cat#F1804; RRID: AB_262044
Mouse anti His-Tag mAb	ABclonal	Cat#AE003; RRID: AB_2728734
Rabbit anti-HA Tag Polyclonal antibody (SG77)	Invitrogen	Cat#71-5500; RRID: AB_2533988
Rabbit anti-GAPDH Polyclonal antibody	Proteintech	Cat#10494-1-AP; RRID: AB_ 2263076
Mouse anti-UBE2T Antibody (OTI2F5)	NovusBio	Cat#NBP2-02965; RRID: AB_3076689
Rabbit anti-Ring1A (D2P4D) mAb	Cell Signaling Technology	Cat#13069S; RRID: AB_2713962
Mouse anti-p53 antibody	Cell Signaling Technology	Cat#48818S; RRID: AB_2713958
Mouse anti-Amylase antibody (G-10)	Santa	Cat#sc-46657; RRID: AB_626668
Rabbit anti-Cytokeratin 19 antibody [EP1580Y]	Abcam	Cat#ab52625; RRID: AB_2281020
Mouse anti-pan-keratin Polyclonal antibody	Cell Signaling Technology	Cat#4545; RRID: AB_490860
Rabbit anti-Ki67 antibody	Abcam	Cat#ab15580; RRID: AB_443209
Rabbit anti-Ki67 antibody	Abcam	Cat#ab16667; RRID: AB_302459
Goat Anti-Rabbit IgG H&L (Alexa Fluor® 488)	Abcam	Cat#ab150077; RRID: AB_2630356
Goat Anti-Mouse IgG H&L (Alexa Fluor® 594)	Abcam	Cat#ab150116; RRID: AB_2650601
Rabbit anti-E2F1 antibody	Cell Signaling Technology	Cat#3742; RRID: AB_2096936
Rabbit anti-Phospho-Rb mAb (Ser807/811)	Cell Signaling Technology	Cat#8516; RRID: AB_11178658
Rabbit anti-Rb antibody [EPR17512]	Abcam	Cat#ab181616; RRID: AB_2848193
Rabbit anti-p21 antibody	Abcam	Cat#ab109520; RRID: AB_10860537
Rabbit anti-Ras (G12D Mutant) Recombinant Monoclonal Antibody (HL10)	Invitrogen	Cat#MA5-36256; RRID: AB_2890403
Rabbit (DA1E) mAb IgG	Cell Signaling Technology	Cat#3900; RRID: AB_1550038
Rabbit anti-UBE2T/HSPC150 Polyclonal antibody	Proteintech	Cat#10105-2-AP; RRID: AB_2211478
Rabbit anti-Ras (mutated G12D) antibody	Abcam	Cat#ab221163; RRID: AB_2877649
Rabbit anti-RPLP0 antibody [EP15646]	Abcam	Cat#ab192866; RRID: AB_2814809
Rabbit anti-Phospho-p44/42 MAPK (Erk1/2) (Thr202/Tyr204) Antibody	Cell Signaling Technology	Cat#9101; RRID: AB_331646
Rabbit anti-ERK1/2 Polyclonal antibody	Proteintech	Cat#11257-1-AP; RRID: AB_2139822
Goat Anti-Mouse IgG (H + L) HRP	Bioworld	Cat#BS12478; RRID: AB_2773727
Goat Anti-Rabbit IgG (H + L) HRP	Bioworld	Cat#BS13278; RRID: AB_2773728

**Bacterial and virus strains**		

DH5α Chemically Competent Cell	AngYuBio	Cat#G6016
SgRNA of UBE2T -#1 (GAGCTCGCAGGTCATCCATT)	This manuscript	N/A
SgRNA of UBE2T -#2 (CATCCAAACATTGATTCTGC)	This manuscript	N/A
SgRNA of UBE2T -#3 (TCTTGCCAACATGTGATGCC)	This manuscript	N/A

**Biological samples**		

Human PDAC tissue	This manuscript	N/A
Patient-derived xenografts (PDX)	This manuscript	N/A
Genetically engineered mice pancreas sample	This manuscript	N/A
KPC/UKPC allografts	This manuscript	N/A
AsPC-1-derived xenografts	This manuscript	N/A

**Chemicals, peptides, and recombinant proteins**		

Ulixertinib	MedChemExpress	Cat#HY-15816
RRx-001	MedChemExpress	Cat#HY-16438
PFK-158	MedChemExpress	Cat#HY-12203
PKM2-IN-1	MedChemExpress	Cat#HY-103617
CPI-613	MedChemExpress	Cat#HY-15453
Palbociclib	MedChemExpress	Cat# HY-50767
Pentagalloylglucose	MedChemExpress	Cat#HY-N0527
MRTX1133	MedChemExpress	Cat#HY-134813
DAPI	Solarbio	Cat#C0060
Cell Counting Kit-8 (CCK-8)	Selleck	Cat#B34302
Basement Membrane Matrix High Concentration	Corning	Cat#354248
Advanced DMEM/F12	Gibco	Cat#12634010
B27 Supplement (50x)	Gibco	Cat#17504044
N-Acetylcysteine	Sigma	Cat#A9165
Recombinant murine EGF	Peprotech	Cat#315-09
Y-27632	MedChemExpress	Cat#HY-10071
TrypLE™ Express Enzyme (1X)	Gibco	Cat#12605028
Dimethyl sulfoxide	MedChemExpress	Cat#HY-Y0320
TBS	Servicebio	Cat#G0001
PBS	Servicebio	Cat#G0002
Protein Phosphatase Inhibitor (All-in-one,100x)	Solarbio	Cat#P1260
Trypsin-EDTA (0.25%)	Gibco	Cat#25200072
Dulbecco’s modified Eagle’s medium (DMEM)	Gibco	Cat#C11995500BT
RPMI 1640 medium	Gibco	Cat#31870082
GlutaMAX™ Supplement	Gibco	Cat#35050061
HEPES(1M)	Gibco	Cat#15630080
Puromycin	Invitrogen	Cat#A1113803
Fetal bovine Serum	Cell-Box	Cat#CF-01S
Phenylmethylsulfonyl fluoride (PMSF)	Beyotime	Cat#ST505
Penicillin-Streptomycin Liquid	Solarbio	Cat#P1400
Hygromycin B	Gibco	Cat#10687010
Geneticin	Gibco	Cat#10131027
Collagenase from Clostridium histolyticum (Type XI)	Sigma	Cat#C7657
Cultrex UltiMatrix Reduced Growth Factor Basement Membrane Extract	Biotechne	Cat#BME001
Human Pancreatic Cancer Organoid Kit	BioGenous	Cat#K2101-PC
Gastrin I (human)	Biotechne	Cat#3006
Recombinant Human FGF-10	Peprotech	Cat#100-26
Nicotinamide	MedChemExpress	Cat#HY-B0150
A 83-01	MedChemExpress	Cat#HY-10432
Prostaglandin E2	MedChemExpress	Cat#HY-101952
Organoid Recovery Solution	BioGenous	Cat#E238006
RB1 Fusion Protein	Proteintech	Cat#Ag11211
G6PD Fusion Protein	Proteintech	Cat#Ag21862
Human Cellular tumor antigen P53/TP53	MedChemExpress	Cat#HY-P72257
Recombinant human E2F1 protein	Abcam	Cat#ab82207
D-Glucose (U-^13^C₆)	Cambridge Isotope Laboratories	Cat#CLM-1396
D-Glucose (U-^13^C_1,2_)	Sigma	Cat#453188
F-127	Sigma	Cat#P2443

**Critical commercial assays**		

Lipo2000™ Transfection reagent Product	Invitrogen	Cat#11668019
PEI 40K Transfection Reagent	Servicebio	Cat#G1802
Agarose gel DNA recovery kit	TIANGEN	Cat#DP219
Plasmid Small Extraction Kit	TIANGEN	Cat#DP103
BCA Protein Assay Kit	Solarbio	Cat#PC0020
Alcian blue Stain Kit	Solarbio	Cat#G1560
Total RNA Extraction Reagent	Tiangen	Cat#DP451
Hematoxylin-Eosin (HE) Stain Kit	Solarbio	Cat#G1120
CellTiter-Glo® 3D Cell Viability Assay	Promega	Cat#G9682
Dual-Luciferase® Reporter Assay System	Promega	Cat#E1910
NADP/NADPH-Glo™ Assays	Promega	Cat#G9082
TIANamp Genomic DNA Kit	TIANGEN	Cat#DP304
DNA pulldown kit	BersinBio	Cat#Bes5004
Spectrum™ Labs Spectra/Por™ 6 1000 D MWCO Standard RC Pre-wetted Dialysis Kits	Fisher Scientific	Cat#08-700-197
His-Tag Protein Labeling Kit (His-Tag RED Channel)	NanoTemper Technologies	Cat#MO-L018

**Deposited data**		

TCGA-PAAD dataset	TCGA	https://portal.gdc.cancer.gov/projects/TCGA-PAAD
Raw immunoblotting data	Mendeley Data	https://doi.org/10.17632/z6s7vb8d77.1
Metabolomic data	METASPACE	https://metaspace2020.eu/project/jiao-2024
RNA-sequencing of pancreatic cancer organoids	NCBI Sequence Read Archive (SRA) data	PRJNA1201558

**Experimental models: Cell lines**		

Human: HEK-293T	Cell Bank of the Chinese Academy of Sciences	Cat#SCSP-502
Human: PANC-1	Cell Bank of the Chinese Academy of Sciences	Cat#SCSP-535
Human: AsPC-1	Pricella	Cat#CL-0027
Mouse: L-WRN	Pricella	Cat#CL-0658
Human: BxPC-3	Pricella	Cat#CL-0042
Patient-derived organoids (PDO)	This manuscript	N/A
Mouse-derived organoids	This manuscript	N/A

**Experimental models: Organisms/strains**		

Mouse: C57BL/6JGpt	Gempharmatech	Cat#N000013; RRID: IMSR_GPT:N000013
Mouse: NOD/ShiLtJGpt-*Prkdc*^em26Cd52^*Il2rg*^em26Cd22^/Gpt(NCG)	Gempharmatech	Cat#T001475; RRID: IMSR_GPT:T001475
Mouse: C57BL/6Smoc-*Kras*^em4(LSL−G12D)Smoc^	Shanghai Model Organisms Center	Cat#NM-KI-190003; RRID: IMSR_NM-KI-190003
Mouse: C57BL/6Smoc-*Ube2t*^tm1(flox)Smoc^	Shanghai Model Organisms Center	Cat#NM-CKO-2115005; RRID: IMSR_NM-CKO-2115005
Mouse: C57BL/6.FVB-Tg(Pdx1-cre)6Tuv/J	Jackson Laboratory	Cat#014647; RRID: IMSR_JAX:014647
Mouse: C57BL/6Smoc-Trp53^tm(LSL−R172H)Smoc^	Shanghai Model Organisms Center	Cat#NM-KI-220071; RRID: IMSR_NM-KI-220071

**Oligonucleotides**		

GenOFF st-h-E2F1_001	Ribobio	Cat#stB0001999A-1-5
GenOFF st-h-E2F1_002	Ribobio	Cat#stB0001999B-1-5
GenOFF st-h-TP53_001	Ribobio	Cat#stB0002017A-1-5
GenOFF st-h-TP53_002	Ribobio	Cat#stB0002017B-1-5
GenOFF st-h-RB1_001	Ribobio	Cat#stB0002011A-1-5
GenOFF st-h-RB1_002	Ribobio	Cat#stB0002011B-1-5
siR Transfect Control	Ribobio	Cat#siT0000001-1-5

**Recombinant DNA**		

pRK5-HA-ubiquitin	This manuscript	N/A
pRK5-UBE2T	This manuscript	N/A
pRK5-RING1	This manuscript	N/A
WT/deletion-mutant/site-mutant FLAG-p53 plasmids	This manuscript	N/A
pRK5-Flag-KRAS^G12D^	This manuscript	N/A
pRK5-Flag-G6PD	This manuscript	N/A
pRK5-Flag-E2F1	This manuscript	N/A
pRK5-HA-E2F1	This manuscript	N/A
pRK5-HA-RING1	This manuscript	N/A
Dual-luciferase vector pGL-4.10	hedgehogBio	HH-LUC-043
pCMV3-His-Rb	Sino Biological	HG10137-NH

**Software and algorithms**		

GraphPad Prism 10.1.2	GraphPad Software	https://www.graphpad.com/
Snapgene	Snapgene by Dotmatics	https://www.snapgene.com/
R 4.3.3	Institute for Statistics and Mathematics	https://www.r-project.org/
Molecular Operating Environment (MOE, version 2020.0901)	Chemical Computing Group ULC, Canada	https://www.chemcomp.com/
SPSS 27.0	International Business Machines Corporation	https://www.ibm.com/
GSEA 4.3.2	Mootha, Lindgren et al.	https://www.gsea-msigdb.org/
Combenefit	Cancer Research UK Cambridge Institute	https://www.cruk.cam.ac.uk/
SynergyFinder web application (version 3.0)	Institute for Molecular Medicine Finland (FIMM)	https://synergyfinder.fimm.fi/

### Experimental model and study participant details

#### Cell lines

HEK-293T and PANC-1 were obtained from the cell bank of the Chinese Academy of Sciences (Shanghai, China), while BxPC-3, AsPC-1 and L-WRN were purchased from Wuhan Pricella Biotechnology Co., Ltd. (Hubei, China). PANC-1, HEK-293T and L-WRN cell lines were cultured with Dulbecco’s Modified Eagle’s Medium (DMEM) and BxPC-3 and AsPC-1 was cultured with Roswell Park Memorial Institute (RPMI) 1640 medium, all supplemented with 10% fetal bovine serum and 1% penicillin-streptomycin solution at 37°C in a 5% CO_2_ atmosphere. Additionally, L-WRN cells received an additional supplement of 0.5 mg/mL hygromycin B and 0.5 mg/mL G-418 for positive screening. When L-WRN cells reached 100% confluence, advanced DMEM/F12 (Gibco, #12634010) was added to prepare L-WRN cell-conditioned medium. The conditioned medium, containing Wnt, R-spondin and Noggin, was collected every 24 h and replaced with fresh advanced DMEM/F12 for three consecutive times. Short Tandem Repeat (STR) profiling was performed to confirm the identity of all cell lines.

#### Animal models

All animal experiments adhered to the ethical standards outlined by the Animal Ethics Committee of the Second Hospital of Lanzhou University (approval number: D2023-485). Genetically engineered mice (GEM) harboring the *LSL-Kras*^*G12D/+*^ or *Ube2t*^*flox/flox*^ mutations (Shanghai Model Organisms Center, Inc., China) and Pdx1-Cre (Jackson Laboratory, USA) were interbred to generate offspring with both *LSL-Kras*^*G12D/+*^ and Pdx1-Cre (KC), as well as *Ube2t*^*flox/flox*^-KC (*Ube2t*^−/−^KC, UKC). The breeding protocols to obtain *LSL-Kras*^*G12D/+*^, *LSL-Trp53*^*R172H/+*^, *Pdx1-Cre* (KPC) and *Ube2t*^*flox/flox*^-KPC (*Ube2t*^−/−^KPC, UKPC) mice have been described previously.^30^

For the subcutaneous transplantation model, KPC or UKPC pancreatic lesion tissue or patient-derived pancreatic cancer specimens were minced to approximately 3 mm^3^, wrapped in a high-concentration basement membrane matrix (Corning, #354248), and subcutaneously implanted into the flank of 6–8 week-old C57BL/6JGpt (Gempharmatech, China, #N000013) or NOD/ShiLtJGpt-*Prkdc*^em26Cd52^*Il2rg*^em26Cd22^/Gpt mice (Gempharmatech, China, #T001475) (patient-derived xenograft, PDX), respectively. Once the mouse grafts reached approximately 1000 mm^3^ in size, tumor tissues were harvested and implanted into the axillary region of the subsequent generation of mice. This iterative process continued until the third generation, at which point further experiments were initiated.

To induce MRTX1133-resistant models, MRTX1133 (MCE, #HY-134813) was administered intraperitoneally at a dosage of 30 mg/kg/day when the tumor volume reached approximately 150 mm^3^, with daily measurements thereafter. Over time, tumors transitioned from initial responsiveness to MRTX1133 treatment to developing tolerance. Upon tumor regrowth, the MRTX1133 dosage was increased to 40 mg/kg/day. As resistance developed further, dosages were increased incrementally until mice exhibited tolerance to MRTX1133 at a dosage of 60 mg/kg/day. Eventually, fresh tumors were collected when tumor volumes reached approximately 1000 mm^3^ and transplanted into the axillary region of mice for subsequent experiments.

#### Organoids construction

KC/UKC organoids were derived from 8-month-old KC/UKC mice and KPC/UKPC organoids were generated from 20-week-old KPC/UKPC mice with palpable pancreatic tumor. This experiment adhered to the ethical standards outlined by the Animal Ethics Committee of the Second Hospital of Lanzhou University (approval number: D2022-312). Human PDAC organoids were obtained from surgically resected PDAC tissues. Human organoids experiments were approved by the Medical Ethics Committee of Lanzhou University Second Hospital and conducted in accordance with the Declaration of Helsinki (approval number: 2022A-454). Written informed consent was obtained from all patients. Tumor samples were processed to remove excess and necrotic tissues, followed by digestion with collagenase XI (Sigma, #C7657, 1 mg/mL) to isolate tumor stem cells. After filtration, the isolated tumor stem cells were suspended in human washing medium [10% fetal bovine serum, 1% penicillin-streptomycin, 2 mM GlutaMAX (Gibco, #35050061), 10 μM HEPES (Gibco, #15630080) in advanced DMEM/F12] and mixed with basement membrane matrix hydrogel (Biotechne, #BME001). The mixture was then plated in cell culture plates. The corresponding human (bioGenous, #K2101-PC) or mouse [50% L-WRN cell conditioned medium, 2 mM GlutaMAX, 10 μM HEPES, 1× B27 (Gibco, #17504044), 1.25 mM N-acetylcysteine (Sigma, #A9165), 10 nM Gastrin Ⅰ (Biotechne, #3006), 50 ng/mL EGF (Peprotech, #315-09), 100 ng/mL FGF10 (Peprotech, #100-26), 10 mM Nicotinamide (MCE, #HY-B0150), 500 nM A83-01 (MCE, #HY-10432), 10.5 μM Y-27632 (MCE, #HY-10071), 1 μM Prostaglandin E2 (MCE, #HY-101952) in advanced DMEM/F12] pancreatic cancer organoid culture medium was added for maintenance culture. Further experiments should commence once the organoids have been successfully passaged to the third generation.

#### Patient samples

This study collected clinical data of 160 patients with pancreatic cancer for expression and survival analysis. The cohort comprised 99 males and 61 females, with ages ranging from 31 to 81 years. All patients were in generally good health aside from their cancer diagnosis and had not received preoperative radiotherapy, chemotherapy, or immunotherapy. Tumor sample from one patient with wild-type KRAS was used to develop organoids and another 5 PC specimens with KRAS^G12D^ mutation were obtained to establish organoids and xenograft models. The research received approval from the Medical Ethics Committee of Lanzhou University Second Hospital and complied with all ethical regulations (approval number: 2022A-133). Informed written consent was obtained from all participants.

### Method details

#### Animal study

Pancreatic tissues from 2-, 4-, 6-, 8-, 10-, and 12-month-old KC and age-matched UKC mice were harvested and fixed in 4% paraformaldehyde for subsequent paraffin embedding. Histological staining was used to assess the neoplastic area and type. The pancreases of 20-week-old KPC and UKPC mice were examined to evaluate pancreatic lesion characteristics, whereas liver tissues from 24-week-old KPC and UKPC mice were analyzed to quantify liver metastases. The OS of KPC/UKPC mice with spontaneous pancreatic tumorigenesis was recorded to assess the impact of UBE2T on long-term survival. Additionally, the KPC and UKPC-GDA models were employed to observe the impact of UBE2T deficiency on short-term tumor progression. Tumor volume was monitored every three days starting from day 6 post-tumor transplantation. The experiment concluded when the tumor volume of any mouse in the two groups reached approximately 1500 mm^3^.

For drug intervention studies, RRx-001 (MCE, #HY-16438) was intraperitoneally administered every other day at a dosage of 5 mg/kg, whereas PGG (MCE, #HY-N0527) was orally administered at a dosage of 40 mg/kg/day. MRTX1133@F127-PGG (MFP) was intraperitoneally administered at doses of 2, 5, and 10 mg/kg, respectively. Upon reaching a tumor volume of approximately 150 mm^3^, mice were randomly assigned to treatment groups. Subsequently, tumor volume and body weight were measured every three days following the initiation of drug treatment. The experiment concluded when the tumor volume of any mouse in all groups reached approximately 1500 mm^3^. All transplanted tumors were excised, weighed, photographed, and fixed for further analysis.

To evaluate the long-term effects of the administered drugs on survival, survival time was recorded, and survival curves were plotted. The 5 and 10 mg/kg doses of MFP were identified as effective therapeutic doses for PDX- and MRTX1133-resistant KPC-allografts and PDX mice. A tumor volume of approximately 1500 mm^3^ was considered equivalent to death, with an OS time of 120 days marking the end of the experiment. PFS was defined as the time taken for the tumor volume to reach 200% of the baseline. Tumor response was determined by comparing the tumor volume change at the endpoint with its baseline: tumor volume change = 100% × ((V_endpoint_ – V_initial_)/V_initial_). The criteria for response were adapted from the RECIST criteria and defined as follows: mCR, tumor volume change < −80%; mPR, tumor volume change < −30%; mSD, tumor volume change <30%; mPD, not otherwise categorized. Mice that were sacrificed owing to adverse events before completing the 14-day trial were excluded from the dataset.

To assess the acute toxicity of MFP, eight-week-old female KM mice were purchase from Lanzhou Veterinary Research Institute, Chinese Academy of Agricultural Sciences, and randomly assigned to two groups (*n* = 6 mice/group). The treatment group received the maximum dosage (10 mg/kg/day) employed in this study, while the control group was administered an equal volume of solvent. Following administration, all mice underwent food and water intake tests, as well as body weight, were recorded every two days. The experiment concluded after 14 days, at which point all mice were anesthetized and subjected to formaldehyde perfusion for organ collection. Subsequently, the organs were evaluated for damage following hematoxylin and eosin staining.

#### Metabolic flux analysis

Human PDAC organoids harboring KRAS^WT^ or KRAS^G12D^ mutations (overexpression) were cultured in medium containing glucose labeled with [U]-^13^C_6_ (Cambridge Isotope Laboratories, #CLM-1396) or [U]^13^C_1,2_ (Sigma, #453188) for 24 h. Subsequently, the medium was discarded, and the organoids were retrieved using an organoid recovery solution (bioGenous, #E238006). After quick freezing in liquid nitrogen, precooled 80% methanol was added and transported on dry ice. Metabolites were detected using the Metabo-Profile (Shanghai, China). The organoid samples were gradually thawed in an ice bath and ultrasonically lysed. Following centrifugation and concentration, the supernatant was subjected to ultra-high-pressure liquid chromatography-triple quadrupole mass spectrometry (ACQUITY-UPLC/Xevo TQ-S, Waters, USA). Peak extraction, integration, identification, and quantitative analysis of each metabolite were performed using MassLynx software (V4.1, Waters, USA). Subsequent statistical analysis was performed using the free, open-source R language (V4.1.1). Dynamic changes in downstream marker metabolites were indicative of alterations in metabolic pathway flow.

#### Central carbon metabolite analysis

Human PDAC organoids with different genotypes underwent analysis using high-performance ion exchange chromatography-tandem mass spectrometry (HPIC-MS/MS) conducted by BIOTREE (Shanghai, China). The organoid samples were treated with precooled MeOH/H_2_O (3/1, v/v), vortexed, subjected to freeze-thaw cycles and sonication, and then incubated at −40°C. After centrifugation, the supernatant was collected and dried. The dried samples were reconstituted with purified water, filtered, and transferred for HPIC-MS/MS analysis. A standard solution containing metabolites was prepared and analyzed using HPIC-MS/MS to establish calibration curves, which were used to quantify metabolite levels. HPIC separation was conducted using a Dionex ICS-6000 HPIC System equipped with AS11-HC and AG11-HC columns (Thermo Fisher Scientific, China). Mobile phase A consisted of 100 mM NaOH in water, while mobile phase B was ultrapure water. An additional pumping system supplied a solvent of 2 mM acetic acid in methanol, mixed with effluent before entering the electrospray ionization (ESI) source at a flow rate of 0.15 mL/min. The column temperature was set to 30°C, with an auto-sampler temperature of 4°C and an injection volume of 5 μL. Mass spectrometry analysis was performed using a 6500 QTRAP (AB SCIEX) with an ESI interface. Multiple reaction monitoring (MRM) parameters were optimized using flow injection analysis with standard solutions, selecting the most sensitive transitions for quantitative monitoring and additional transitions as qualifiers for verifying analyte identity.

#### Spatial metabolomics analysis

Pancreatic tissues of 8-month-old KC (*n* = 3) and UKC (*n* = 3) mice were removed and embedded with tissue freezing medium (Leica Microsystem, Germany), then sent to Oebiotech (Shanghai, China) for spatial metabolomics analysis. The embedded samples were cut into consecutive sagittal slices 10 μm about 10 slices by a cryostat microtome (Leica CM 1950, Leica Microsystem, Germany) and were thaw-mounted on positive charge desorption plate (Thermo Scientific, USA). Mass spectrometry imaging (MSI) analysis was carried out with an AFADESI-MSI platform (Beijing Victor Technology, Beijing, China) in tandem with a Q-Orbitrap mass spectrometer (Q Exactive, Thermo Scientific, USA). The solvent formula was acetonitrile (ACN)/H_2_O (8:2) at negative mode and ACN/H_2_O (8:2, 0.1% FA (formylic acid (HCOOH))) at positive mode and the solvent flow rate was 5 μL/min, the transporting gas flow rate was 45 L/min, the spray voltage was set at 7 kV, and the distance between the sample surface and the sprayer was 3 mm as was the distance from the sprayer to the ion transporting tube. The MS resolution was set at 70,000, the mass range was 70–1000 Da, the automated gain control (AGC) target was 2E6, the maximum injection time was set to 200 ms, the S-lens voltage was 55 V, and the capillary temperature was 350°C. The MSI experiment was carried out with a constant rate of 0.2 mm/s continuously scanning the surface of the sample section in the x direction and a 50 μm (KC vs. UKC) vertical step in the y direction.

The raw data were viewed and analyzed by MSiReader software (an open-source interface on MATLAB platform) and ion image reconstructions were carried out using the Cardinal software package after background subtraction. All MS images were normalized using total ion count normalization (TIC) in each pixel. Region-specific MS profiles were precisely extracted by matching high-spatial resolution HE images. The discriminating endogenous molecules of different tissue microregions were screened by a supervised statistical analytical method: orthogonal partial least squares discrimination analysis (OPLS-DA). Variable Importance of Projection (VIP) values obtained from the OPLS-DA model were used to rank the overall contribution of each variable to group discrimination. The VIP value reflects the importance degree on the classification of sample categories with respect to the first two principal components of the OPLS-DA model, which indicates that this variable has a significant effect if the VIP is greater than 1. A two-tailed Student’s T-test was further used to verify whether the metabolites of difference between groups were significant. Differential metabolites were selected with VIP values greater than 1.0 and *p*-values less than 0.05. For the special data structure obtained from the MSI analysis, T-distributed stochastic neighbor embedding (t-SNE) and uniform manifold approximation and projection for dimension reduction (UMAP) on the MS data in each pixel for dimensionality reduction were performed respectively. The Spatial shrunken centroids clustering (SSCC) was applied for MSI data clustering to separate the sample based on the difference’s abundance of ions in each pixels. The ions detected by AFADESI were annotated by the pySM pipeline and an in-house SmetDB database. MSI was used to assess metabolite levels within different pathological microregions. To analyze the distribution of characteristic metabolites across these microregions, we employed SSCC analysis. This approach allowed us to identify and cluster metabolites based on their spatial distribution patterns and abundance profiles. Subsequently, we utilized t-SNE to visualize the SSCC clustering results in a two-dimensional plane. This dimensionality reduction technique facilitated the display of complex high-dimensional data, highlighting the distribution characteristics of the metabolites within the clusters. Clusters 1 through 8 represent groups of metabolites with distinct features.

#### G6PD enzyme activity assay

The organoids were cultured in 96-well plates, and once the ring structure formed, the respective drug was added. Following six days of continuous drug exposure, the levels of NADP and NADPH were measured to determine the relative activity of G6PD using the NADP/NADPH-Glo Assay kit (Promega, #G9082), following the manufacturer’s instructions.

#### Drugs synergy evaluation

According to IC50 values for human or mouse pancreatic cancer organoids and the solubility of RRx-001, PFK-158 (MCE, #HY-12203), PKM2-IN-1(MCE, #HY-103617), CPI-613 (MCE, #HY-15453), MRTX1133 and PGG, six drug concentrations gradients were set for RRx-001, PFK-158, PKM2-IN-1, CPI-613, MRTX1133 and PGG, respectively. The organoids were planted in 96-well plates, and the corresponding drugs were added according to different drug concentration combinations when the obvious ring structure formed. After 6 days of continuous intervention, the viability of the organoids was detected by CellTiter-Glo 3D Cell Viability Assay (Promega, #G9682) under the manufacturer’s instructions. The synergistic therapeutic effect of RRx-001 and MRTX1133, PFK-158 and MRTX1133, PKM2-IN-1 and MRTX1133, CPI613 and MRTX1133, on human and mouse pancreatic cancer organoids were evaluated using Combenefit software and the SynergyFinder web application (version 3.0). The three-dimensional visualization of the synergistic effect was presented by the LOEWE model in Combenefit software. The synergy scores in LOEWE, ZIP, HSA and BLISS models were calculated by SynergyFinder web application.

#### Construction of MRTX1133@F-127-PGG nanoparticles

The MRTX1133@F-127-PGG (MFP) nanoparticles were synthesized through self-assembly, leveraging hydrogen bond interactions between PGG and F127 (Sigma, #P2443), as well as hydrophobic interactions between the polypropylene oxide chain within F127 and MRTX1133. Specifically, F127 (50 mg), PGG (25 mg), and MRTX1133 (25 mg) were independently dissolved in DMSO (5 mL). Subsequently, the solutions were combined and stirred at 25°C overnight. The resulting MRTX1133@F-127-PGG (MFP) nanoparticles were obtained as a white powder after dialyzing against deionized water for 72 h using a dialysis bag (MWCO: 1000 Da, Fisher Scientific, #08-700-197), followed by freeze-drying. Characterization of MRTX1133@F-127-PGG nanoparticles.

The morphologies of MFP were measured by transmission electron microscopy (TEM, Hitachi, Japan). The hydrodynamic diameter of MFP was evaluated by dynamic light scattering (DLS), performed at 25°C with the 90Plus Pals equipment (Brookhaven Instruments Corporation, USA). The stability of MFP particles was measured after storage in PBS for 0 days, 1 day, 3 days, 5 days and 7 days with DLS. The UV-vis absorbance curves of PGG, F-127, MRTX1133 and MFP in DMSO were conducted by UV-vis spectrophotometer (SHIMADZU, Japan). The MRTX1133 content in MFP were determined using UV-vis spectrophotometer with the standard curve of MRTX1133 in DMSO. Fourier transform infrared spectroscopy (FT-IR) spectral analysis was carried out on a Thermo Fisher Nicolet is5 infrared spectrometer (Bruker, Karlsruhe, Germany) in the range between 4000 cm-1 and 400 cm-1. 1H NMR spectra of F127, PGG, MRTX1133 and MFP were recorded on a JEOL ECS (400 M) spectrometer in DMSO-d6. *In vitro* MRTX1133 release of MFP nanoparticles were performed in pH 7.4 PBS and pH 5.0 PBS using dialysis bag (MWCO = 1000 Da) at 37°C in an incubator shaker. Preset amount of MFP nanoparticles solution (10 mL) was transferred into the dialysis bag and immersed in corresponding buffer solution (100 mL). Five mL of the incubated solution was taken out at different time intervals, and the same amount of fresh buffer was added to keep the volume constant. MRTX1133 release profiles were characterized by measuring the UV-vis absorbance of the solutions at 340 nm with the help of a calibration curve of MRTX1133 in the same PBS.

#### TCGA analysis

The counts and clinical data of PAAD were downloaded from TCGA and matched. The difference of *UBE2T* in patient with PDAC harboring KRAS^WT^ or KRAS^G12D^ and the correlation between KRAS^G12D^ and overall survival were analyzed. The c2.cp.kegg_legacy.v2023.2.Hs.symbols.gmt dataset in the Molecular Signature Database (MsigDB) was imported into GSEA 4.3.2 software for KEGG pathway enrichment analysis between KRAS wild-type and G12D mutant PDAC tissues, which was visualized by the ggplot2 and ggridges packages in R-4.3.3.

#### Organoid viability and invasion assay

Details of the organoid viability assay have previously been described.^30^ For organoid invasion assay, organoids were mixed with 50%, 30%, 15% matrigel and seeded in culture plates. After 3 days of culture in Matrigel, organoids images were taken with an OLYMPUS IX53 microscope (Olympus Corporation, Japan) using phase contrast. The number of organoid pseudopods was related to invasiveness.

#### Induction of MRTX1133-resistant AsPC-1 cells

The method for inducing MRTX1133-resistant AsPC-1 cells was based on our previous publication (30). Initially, the induction concentration was set at IC20, with subsequent gradual increases in concentration until MRTX1133-resistant AsPC-1 cell lines exhibiting 5-fold and 10-fold IC50 values were established.

#### Lentiviral infection of organoids

Specific infection methods have been described in our previous study.^30^ Lentivirus of *UBE2T* knockout, UBE2T overexpression and KRAS^G12D^ overexpression were purchased from Shanghai Genechem Co., Ltd (China). The sequences of guide RNAs (SgRNAs) of *UBE2T* were as follows: SgRNA-#1 (GAG CTC GCA GGT CAT CCA TT), SgRNA-#2 (CAT CCA AAC ATT GAT TCT GC) and SgRNA-#3 (TCT TGC CAA CAT GTG ATG CC).

#### Plasmids and small interfering RNAs

Gibson assembly-cloning method was used to generate plasmids in this study. The cDNA sequences of KRAS^G12D^, G6PD, E2F1, GFP genes were delivered into the pRK5-FLAG vector for temporary expression. In addition, the cDNA sequences of E2F1 and RING1 gene were cloned into the pRK5-HA vector. pCMV3-His-Rb (#HG10137-NH) was purchased from Sino Biological Inc. (Beijing, China). pRK5-HA-Ubi, pRK5-*Ring1*, pRK5-*UBE2T*, deletions-mutant p53 plasmids were constructed in our previous article.^30^ The cDNA sequences of six promoter regions of UBE2T were cloned into the dual-luciferase vector pGL4.10-hRluc (hedgehogBio, #HH-LUC-043, Shanghai, China) for dual-luciferase reporter gene assay. GenOFF Small interfering RNAs for *TP*53 (#stB0002017A/B-1-5), E2F1 (#stB0001999 A/B-1-5), Rb (stB0002011A/B-1-5) and corresponding controls (#siT0000001-1-5) were purchased from Guangzhou RiboBio (Guangzhou, China) and transfected according to the manufacturer’s instructions.

#### *In vivo* ubiquitination assay

The indicated plasmids were transfected into HEK-293T cells by Lipofectamine 2000 (Thermo Fisher, #11668019), and subsequent procedures were performed as described in previous article.^53^

#### Dual-luciferase reporter gene (dual-luc) assay

The binding motif of E2F1 and *UBE2T* promoter was predicted by JASPAR database, and six regions were obtained as follows: *-1007 ∼ -1017 bp*, *-973 ∼ -983 bp*, *-876 ∼ -886 bp*, *-839 ∼ -849 bp*, *-828 ∼ -838 bp*, *-820 ∼ -830 bp*. The full-length and six regions of *UBE2T* promotor sequence were cloned into the dual-luciferase vector pGL-4.2.2. HEK-293T cells were seeded in 24-well plates, and then 200 ng plasmids of the dual-luciferase reporter with full-length and six regions of *UBE2T* promotor, pRK5-Flag-KRAS^G12D^ and pRK5-E2F1 were co-transfected using Lipofectamine 2000 when the cells were 70% confluent. After 24 h, firefly luciferase activity and renilla luciferase activity were detected using the Dual-Luciferase Reporter Assay System (Promega, #E1910) according to the manufacturer’s instructions.

#### Immunoprecipitation

The indicated plasmids were transfected into HEK-293T cells, and subsequent procedures were performed as previously described.^53^

#### Quantitative real-time PCR

The specific method was as described in the previous article.^53^ GAPDH (Forward primer: GCA CCG TCA AGG CTG AGA AC, Reverse primer: TGG TGA AGA CGC CAG TGG A) was used as a control to analyze the transcriptional expression of *UBE2T* (Forward primer: ATC CCT CAA CAT CGC AAC TGT, Reverse primer: CAG CCT CTG GTA GAT TAT CAA GC) or E2F1 (Forward primer: CAT CCC AGG AGG TCA CTT CTG, Reverse primer: GAC AAC AGC GGT TCT TGC TC).

#### Genotype identification of KRAS

The genomic DNA of the human PDAC organoids or tissues was extracted using the TIANamp Genomic DNA Kit (TIANGEN, #DP304, Beijing, China), then subjected to PCR amplification. And the sequencing analysis was performed by Tsingke Biotech (Xian, China), and results were read using SnapGene software (SnapGene, China). The forward-primer of KRAS fragment was CTG GTG GAG TAT TTG ATA GTG, and the reverse-primer was CTG TAT CAA AGA ATG GTC CTG.

#### DNA pull down assay

The DNA pull down kit (BersinBio, #Bes5004, Guangzhou, Beijing) was used to find the transcription factors that bind to the *UBE2T* promoter domain. Firstly, DNA probes were designed for the *UBE2T* promoter region and labeled with desulfurized biotin by Sangon Biotech (Shanghai, China). Then the magnetic beads coupled with streptavidin specifically bound to the DNA probe labeled with desulfobiotin to prepare a DNA probe-magnetic bead complex. The nuclear protein of PANC-1 cells was extracted, removed the nucleic acid and incubated with the DNA probe-magnetic bead complex. The potential *UBE2T* transcription factor could specifically bind to the DNA probe. After washing, the non-specific binding protein molecules can be removed. Finally, the DNA probe-protein complex was obtained after elution of streptavidin, and immunoblotting or mass spectrometry was used to identify transcription factors of *UBE2T*.

#### Transcriptomics analysis

The PDAC organoids overexpressing KRAS^G12D^ and harboring wild-type KRAS were subjected to transcriptome detection by BIOTREE (Shanghai, China). Total RNA was extracted using Trizol reagent following the manufacturer’s protocol. High-quality RNA samples (RNA integrity number >7.0) were utilized for sequencing library construction. mRNA was purified using Dynabeads Oligo (dT) (Thermo Fisher, CA, USA) from total RNA and then fragmented using divalent cations under elevated temperature [Magnesium RNA Fragmentation Module (NEB, #e6150, USA) under 94°C 5–7 min]. These fragments were reverse-transcribed to generate cDNA, followed by synthesis of U-labeled second-stranded DNAs. Adapters were ligated to the fragments, which underwent size selection. After PCR amplification, cDNA libraries with an average insert size of 300 ± 50 bp were prepared for Illumina Novaseq 6000 sequencing. After alignment with reference genome and quantification of gene abundance, differential expression analysis was conducted using DESeq2 software between two different groups. The genes with the parameter of false discovery rate (FDR) below 0.05 and absolute fold change ≥2 were considered differentially expressed genes. Differentially expressed genes were subjected to subsequent analysis.

#### Microscale thermophoresis assay

Monolith NT.115 system (NanoTemper Technologies GmbH, Germany) was used to quantify the interaction of E2F1 (Abcam, #ab82207) and Rb (Proteintech, #Ag11211), p53 (MCE, #HY-P72257) and G6PD (Proteintech, #Ag21862), p53 and E2F1. Rb and p53 was fluorescently labeled with His-Tag Labeling Kit RED-tris-NTA 2nd Generation (NanoTemper Technologies, #MO-L018). Different concentrations of G6PD, E2F1, E2F1 were co-incubated respectively with p53, p53, Rb, followed by microscale thermophoresis (MST) analysis. The obtained values were normalized and plotted. Dissociation constants were determined using a one-point model to fit the curve. The details of the MST assay have been described in our previous study.^30^

#### Histopathological staining

Paraffin-embedded tissues were subjected to HE staining, immunofluorescence (IF) staining, immunohistochemical (IHC) staining, and Alcian blue staining according to the methods in our previous study.^30^ For IF staining, the primary antibodies were as follows: rabbit anti-CK19 antibody (1:200, Abcam, #ab52625), mouse anti-amylase antibody (1:200, Santa, #sc-46657), mouse anti-Pan-Keratin antibody (1:200, CST, #4545), rabbit anti-Ki67 antibody (1:200, Abcam, #ab15580/ab16667). For IHC staining, the staining of rabbit IgG (1:200, CST, #3900) as isotype control and used primary antibodies were as follows: rabbit anti-RAS^G12D^ antibody (1:100, Thermo Fisher, #MA5-36256), anti-UBE2T antibody (1:100, NovusBio, #NBP2-02965), anti-Ki67 antibody (1:200, Abcam, #ab15580/ab16667).

#### Immunoblotting

The specific procedure of immunoblotting was as previously described.^53^ The primary antibodies used in the article were as follows: mouse anti-FLAG antibody (1:1000, Sigma, #F1804), rabbit anti-HA antibody (1:1000, Invitrogen, #71–5500), mouse anti-His antibody (1:1000, ABclonal, #AE003), rabbit anti-E2F1 antibody (1:1000, CST, #3742), rabbit anti-p21 (1:1000, Abcam, #ab109520), mouse anti-p53 antibody (1:1000, CST, #48818S), rabbit anti-phospho-Rb (1:1000, CST, #8516), rabbit anti-Rb antibody (1:1000, Abcam, #ab181616), rabbit anti-RING1 (1:1000, CST, #13069S), rabbit anti-UBE2T (1:1000, Proteintech, #10105-2-AP), rabbit anti-RAS^G12D^ antibody (1:1000, Abcam, #ab221163), rabbit anti-GAPDH (1:1000, Proteintech, #10494-1-AP), rabbit anti-RPLP0 (1:1000, Abcam, #ab192866), rabbit anti-phosphor-ERK1/2 antibody (1:1000, CST, #9101), rabbit anti-ERK1/2 antibody (1:1000, Proteintech, #11257-1-AP). The ratio of secondary antibody of goat anti-mouse IgG (Bioworld, #BS12478) or anti-rabbit IgG (Bioworld, #BS13278) was 1:10000.

### Quantification and statistical analysis

Statistical analyses were performed using SPSS 27.0 and GraphPad Prism 10.1.2. Shapiro–Wilk test was used to assess the normality of the data. For normally distributed samples, Student’s t test and one-way ANOVA were used to compare differences between two and multiple groups, respectively. Subsequently, post hoc analysis was conducted using the least significant difference (LSD) method or the Tamhane method for homogeneity and heterogeneity of variances, respectively. Non-normally distributed values were assessed using nonparametric tests. Survival analysis was conducted using Kaplan–Meier curves, and the log rank test was used to assess survival outcomes.

Linear regression analysis was employed to assess the correlation between G6PD activity and the IC50 of MRTX1133 in PDOs. Multivariate Cox models were employed to assess the impact of assumed risk factors on the OS of patients with high RAS^G12D^. The chi-square test was used to evaluate the association and dependence of UBE2T with other assumed risk factors. All tests were two-sided, and statistical significance was set at *p* < 0.05. *In vitro* assays were repeated at least thrice with biological and technical replicates, and 'n' represents the number of independent biological replicates per group.

## Supplemental information

Document S1. Figures S1–S14

Table S1. Raw data of metabolic flux analysis, related to Figures 1, 3, and 6

Document S2. Article plus supplemental information
