# Supplementary material for: KRASG12D-driven pentose phosphate pathway remodeling imparts a targetable vulnerability synergizing with MRTX1133 for durable remissions in PDAC
Source: Cell Rep Med. 2025 Feb 18;6(2):101966. doi: 10.1016/j.xcrm.2025.101966 (PMC11866490; doi:10.1016/j.xcrm.2025.101966)
Supplement: Document S1. Figures S1–S14 [file mmc1.pdf]

Cell Reports Medicine, Volume 6

Supplemental information

KRAS^{G12D}-driven pentose phosphate pathway remodeling imparts a targetable vulnerability synergizing with MRTX1133 for durable remissions in PDAC

Xiangyan Jiang, Tao Wang, Bin Zhao, Haonan Sun, Yuman Dong, Yong Ma, Zhigang Li, Yuxia Wu, Keshen Wang, Xiaoying Guan, Bo Long, Long Qin, Wengui Shi, Lei Shi, Qichen He, Wenbo Liu, Mingdou Li, Lixia Xiao, Chengliang Zhou, Hui Sun, Jing Yang, Junhong Guan, Huinian Zhou, Zeyuan Yu, and Zuoyi Jiao

Supplementary Figures

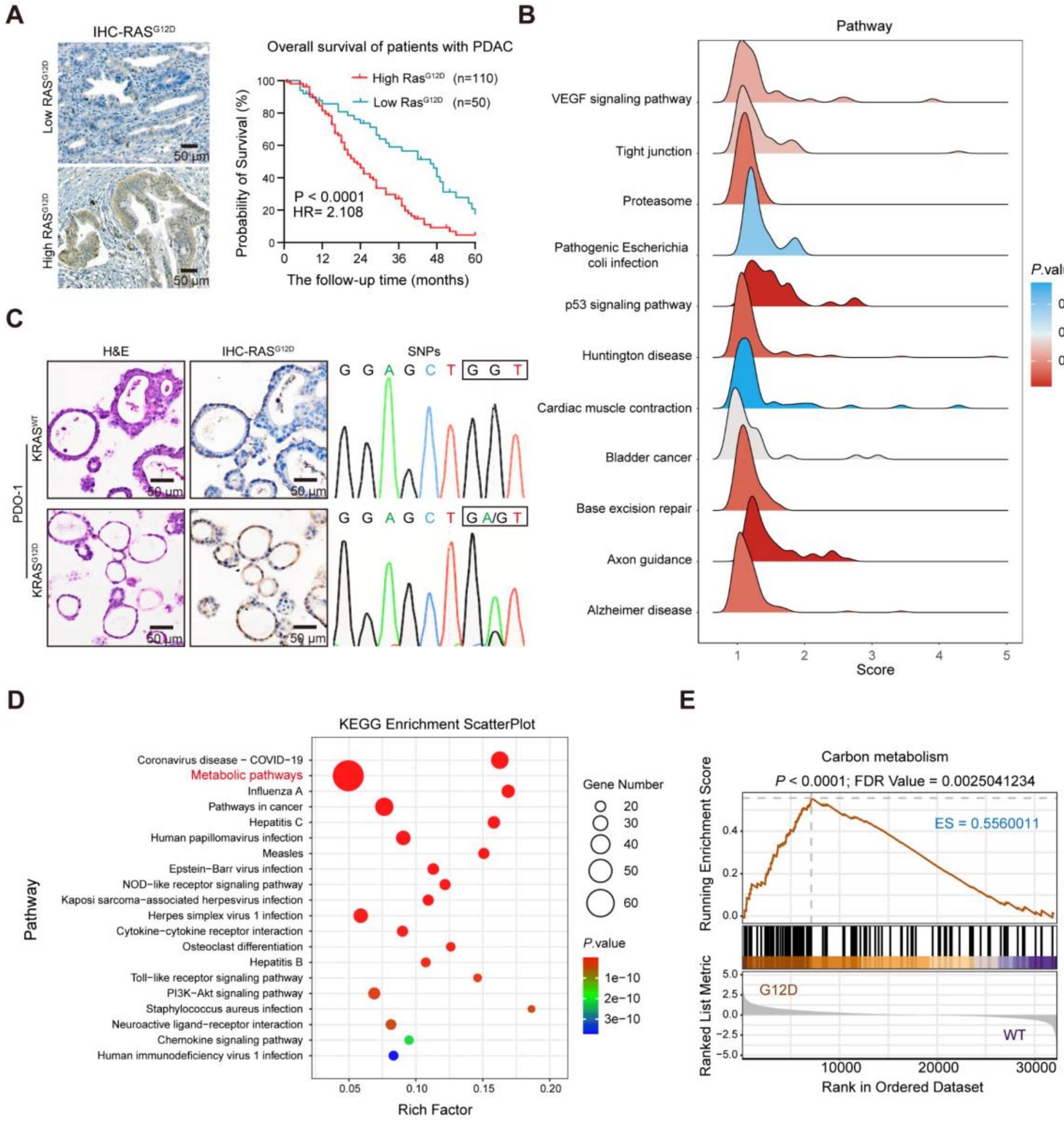

Figure S1. KRAS^{G12D} promotes center carbon metabolism in PDAC. Related to Figure 1.

(A) Representative images of RAS^{G12D} staining in PDAC tissues from patients (left). Kaplan-Meier survival curves with log-rank test for patients stratified by RAS^{G12D} protein levels in our cohort (n = 160) (right).

(B) KEGG analysis for pathway using DEGs from patients harboring KRAS^{WT} and KRAS^{G12D} mutations in TCGA database.

(C) Representative images showing H&E staining, RAS^{G12D} immunohistochemistry, and single nucleotide polymorphism analysis at the KRAS G12 site in organoids with or without KRAS^{G12D} overexpression.

(D) KEGG analysis for pathway using DEGs from KRAS^{WT} and KRAS^{G12D} organoids detected by transcriptomics.

(E) GSEA analysis of center carbon metabolism from KRAS^{WT} and KRAS^{G12D} organoids based on transcriptomics data.

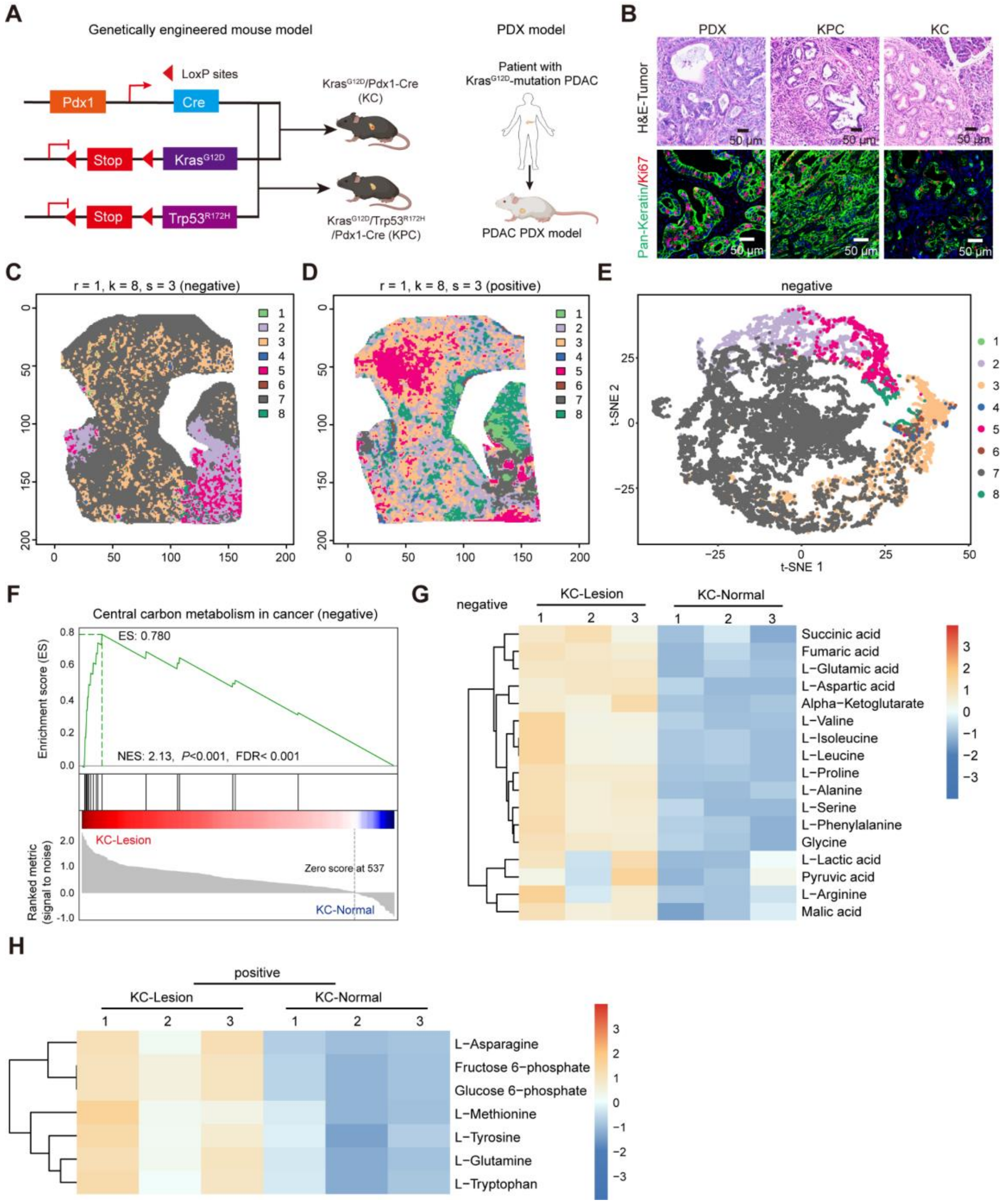

Figure S2. Spatial metabolomics analysis in KRAS^{G12D} PDAC. Related to Figure 1.

(A) Schematic diagram illustrating the construction of KC, KPC, and PDX models.

(B) Representative images of H&E, pan-keratin/Ki67 staining in KC, KPC, and PDX models.

(C and D) Spatial shrunken centroids clustering (SSCC) visualization of spatial metabolomics in negative (C) and positive (D) ionization conditions. Clusters 1 through 8 represent groups of metabolites with distinct characteristics identified through our SSCC analysis.

(E) t-SNE visualization of spatial metabolomics in negative ionization conditions.

(F) GSEA analysis of center carbon metabolism based on spatial metabolomics analysis.

(G and H) Differential metabolites of center carbon metabolism in normal pancreas and lesion tissues detected by spatial metabolomics analysis in negative (G) and positive (H) condition.

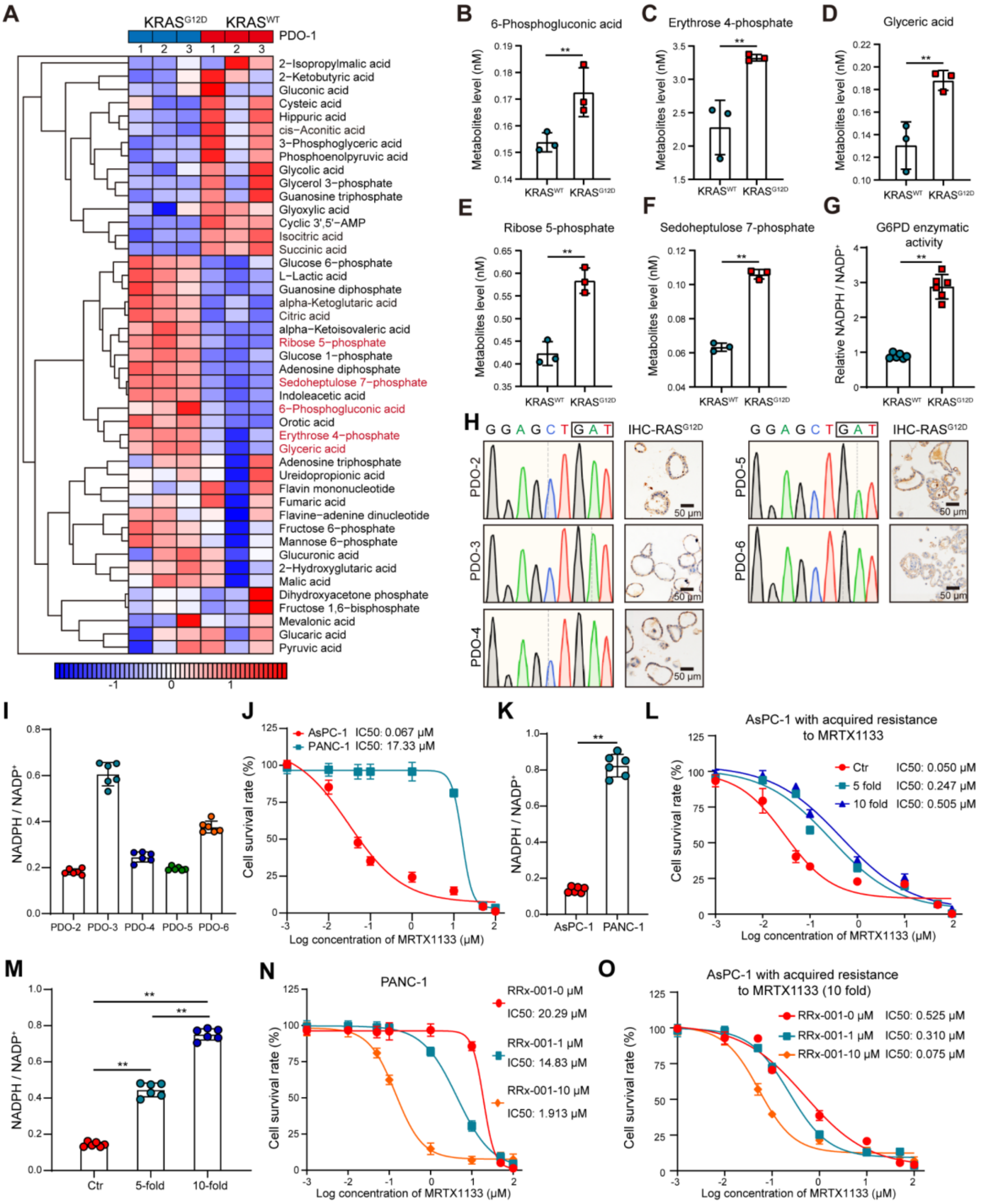

Figure S3. Targeted metabolomics on center carbon metabolism analysis in KRAS^{G12D} and KRAS^{WT} PDAC. Related to Figure 1.

(A) Heatmap showing differential metabolites in center carbon metabolism pathway (n = 3).

(B-F) Level of 6-phosphogluconic acid (B), Erythrose 4-phosphate (C), Glyceric acid (D), Ribose 5-phosphate (E), and Sedoheptulose 7-phosphate (F) in KRAS^{WT} and KRAS^{G12D} organoids based on targeted metabolomics on center carbon metabolism analysis (n = 3).

(G) G6PD enzyme activity detection assessed by NADPH/NADP$^+$ in KRAS^{WT} and KRAS^{G12D} organoids (n = 6).

(H) Representative images showing RAS^{G12D} immunohistochemistry and single nucleotide polymorphism analysis at the KRAS G12 site in PDO-2, 3, 4, 5, and 6.

(I) G6PD enzyme activity detection assessed by NADPH/NADP$^+$ in PDO-2, 3, 4, 5, and 6 (n = 6).

(J) Sensitivity to MRTX1133 in AsPC-1 and PANC-1 cells (n = 6).
(K) G6PD enzyme activity detection assessed by NADPH/$NADP^+$ in AsPC-1 and PANC-1 cells (n = 6).
(L) Sensitivity to MRTX1133 in wild-type, 5-fold, and 10-fold acquired drug-resistance AsPC-1 cells (n = 6).
(M) G6PD enzyme activity detection assessed by NADPH/$NADP^+$ in wild-type and acquired drug-resistance AsPC-1 cells (n = 6).
(N) Sensitivity to MRTX1133 in PANC-1 cells treated with the different-concentration RRX-001 (n = 6).
(O) Sensitivity to MRTX1133 in acquired drug-resistance AsPC-1 cells treated with the different-concentration RRX-001 (n = 6).
Mean ± SD, Student's t test. **$P < 0.01$.

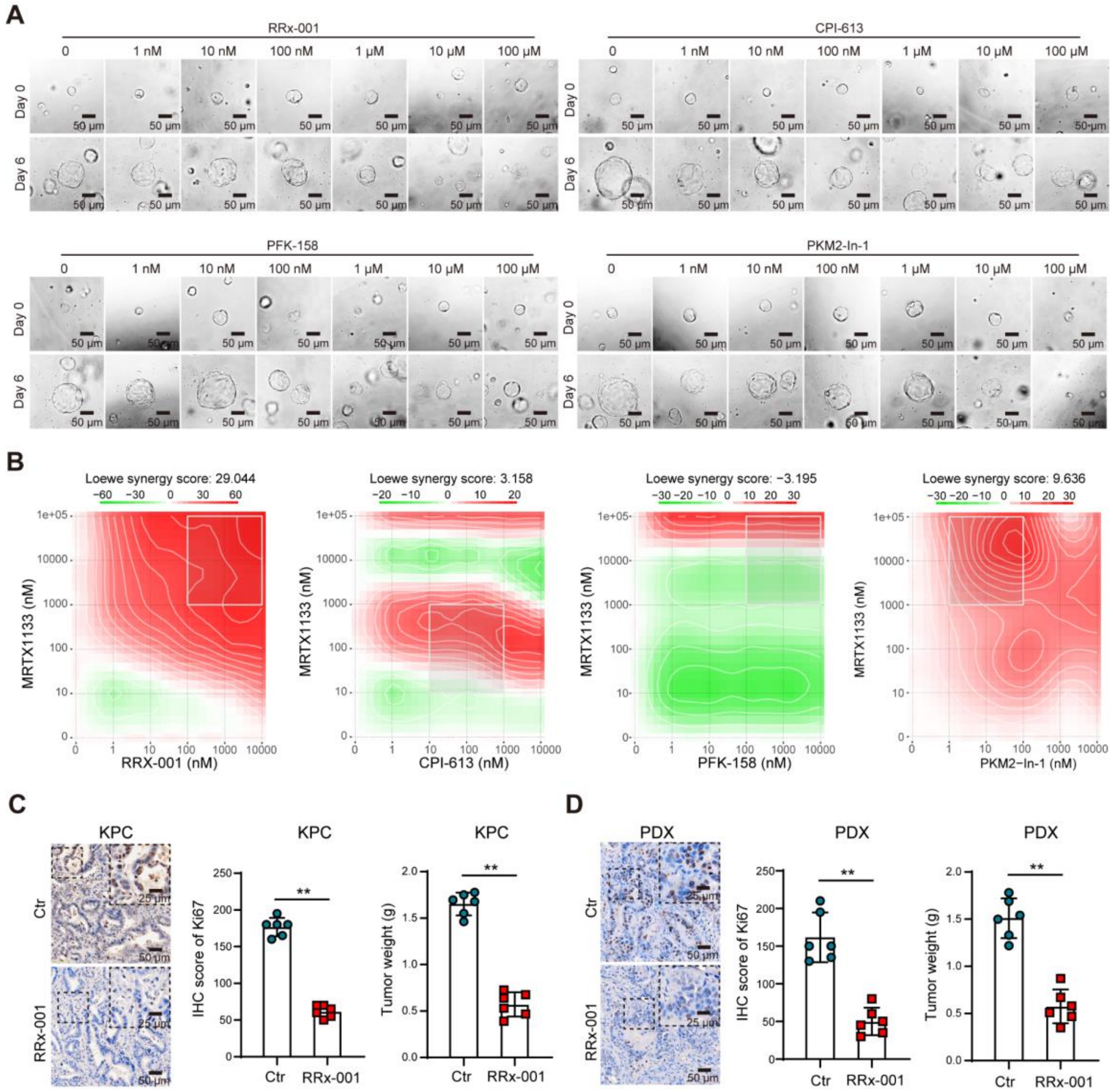

Figure S4. Targeting G6PD-mediated PPP inhibits progression and synergize with MRTX1133. Related to Figure 2.

(A) Representative images of PDO-3 treated with RRx-001, CPI-613, PFK-158, and PKM-In-1.

(B) Synergy analysis of MRTX1133 with RRx-001, CPI-613, PFK-158, and PKM2-In-1 in PDO-3, evaluated using the Loewe model.

(C and D) Representative images of Ki67 staining and quantitation, and tumor weight in KPC allografts (C) and PDX models (D) (n = 6).

Mean ± SD, Student's t test. **P < 0.01.

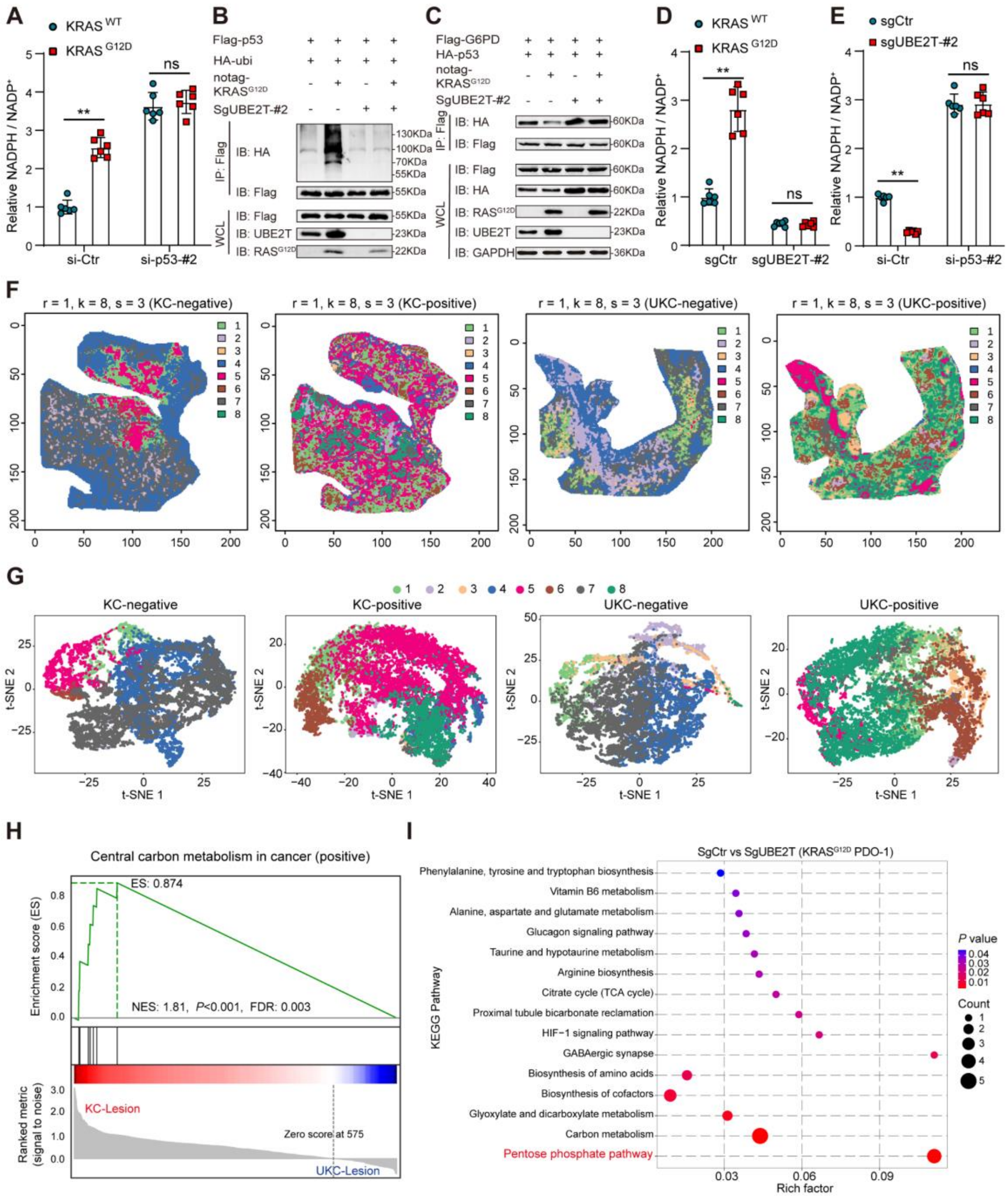

Figure S5. UBE2T is associated with center carbon metabolism. Related to Figure 3.

(A) G6PD enzyme activity detection assessed by NADPH/NADP$^+$ in KRAS^{WT} or KRAS^{G12D} PDO-1 with or without *Tp53* knockdown (siRNA-#2) (n = 6).

(B) Ubiquitination assay illustrating that the degree of p53 ubiquitination in Control (sgCtr) or *UBE2T*-knockout (sgUBE2T, sgRNA-#2) BxPC-3 cells expressing the indicated plasmids.

(C) Co-immunoprecipitation (IP) assays reveal the interaction between p53 and G6PD in sgCtr or SgUBE2T (sgRNA-#2) BxPC-3 cells coexpressing the indicated plasmids.

(D) G6PD enzyme activity in KRAS^{WT} or KRAS^{G12D} PDO-1 with or without *UBE2T* deletion (sgRNA-#2) (n = 6).

(E) G6PD enzyme activity in SgCtr or SgUBE2T PDO-3 with or without *Tp53* knockdown (si-RNA-#2) (n = 6).

(F) SSCC visualization of spatial metabolomics in KC and UKC lesion tissues.

(G) t-SNE visualization of spatial metabolomics in KC and UKC lesion tissues. Clusters 1 through 8 represent groups of metabolites with distinct characteristics identified through our SSCC analysis.

(H) GSEA analysis of center carbon metabolism in KC and UKC lesion tissues based on spatial metabolomics analysis.

(I) KEGG analysis for pathway using DEGs from $KRAS^{G12D}$ organoids with or without *UBE2T* knockout based on targeted metabolomics on center carbon metabolism.

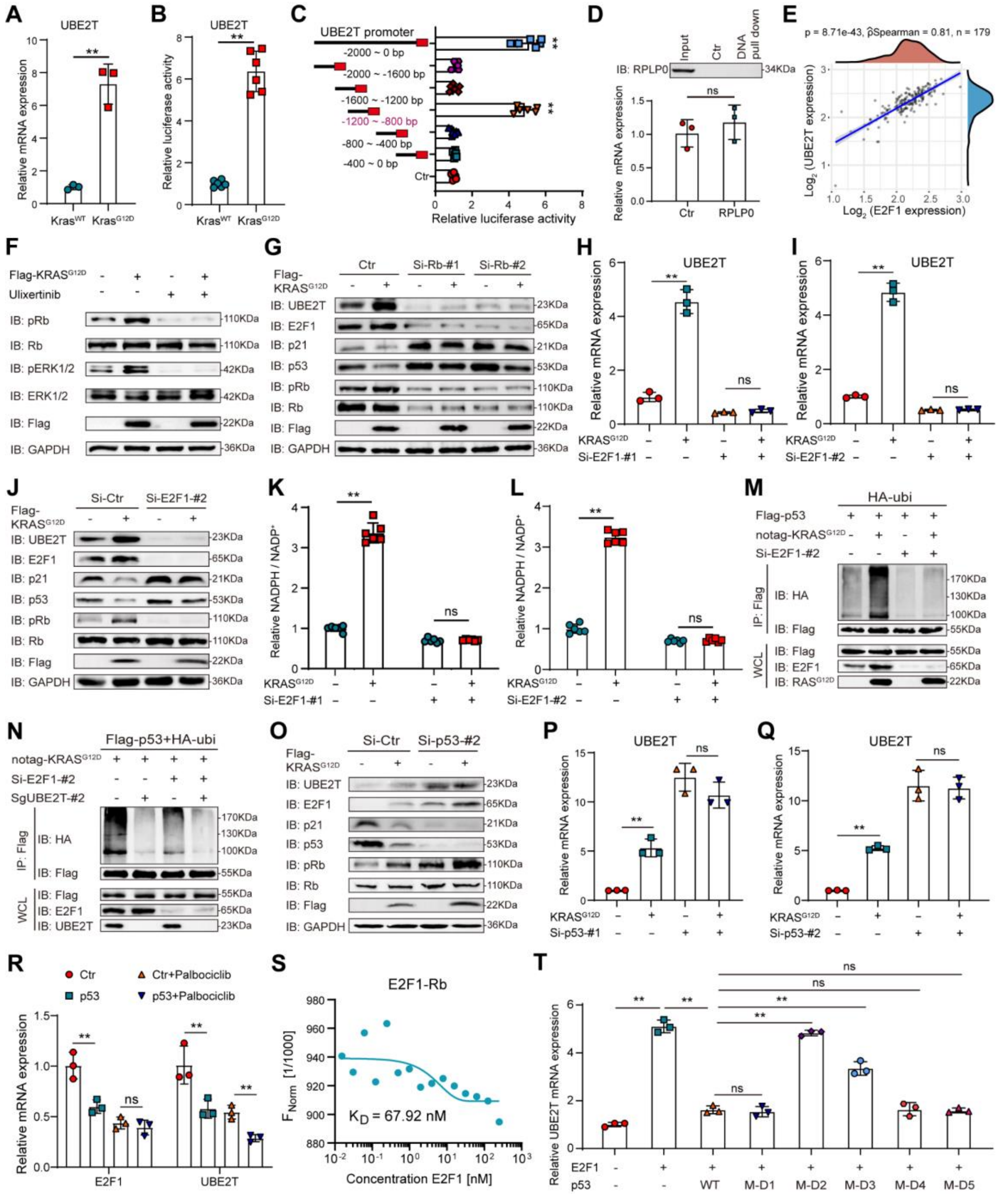

Figure S6. KRAS^{G12D} promotes UBE2T transcription by p53 ubiquitination-mediated feedback loops. Related to Figure 4.

(A and B) Quantitative real-time PCR (qRT-PCR) (n = 3) (A) and Dual-luciferase gene reporter assays (n = 6) (B) show the mRNA expression and transcription of the *UBE2T* with or without KRAS^{G12D} overexpression.

(C) Dual-luciferase gene reporter assays show the transcriptional activities of the indicated *UBE2T* promoter with or without KRAS^{G12D} overexpression.

(D) DNA pull down assay shows the interaction of RPLP0 with *UBE2T* promoter (top). qRT-PCR assays show the mRNA expression of *UBE2T* with or without RPLP0 overexpression (bottom) (n = 3).

(E) The mRNA correlation between E2F1 and *UBE2T* in patients with PDAC from TCGA database.

(F) Immunoblotting (IB) analysis with the indicated antibodies in Control or KRAS^{G12D}-overexpresed

BxPC-3 cells treated with or without ulixertinib (the ERK1/2 phosphorylation inhibitor).
(G) IB analysis with the indicated antibodies in Control or $KRAS^{G12D}$-overexpresed BxPC-3 cells with or without Rb knockdown.
(H and I) qRT-PCR assays show the mRNA expression of *UBE2T* in BxPC-3 cells with or without $KRAS^{G12D}$ expression and/or E2F1 knockdown (H: siRNA-#1. I: siRNA-#2) (n = 3).
(J) IB analysis with the indicated antibodies in Control or $KRAS^{G12D}$-overexpreesed BxPC-3 cells with or without E2F1 knockdown (siRNA-#2).
(K and L) G6PD enzyme activity detection assessed by NADPH/$NADP^+$ in BxPC-3 cells with or without $KRAS^{G12D}$ expression and/or E2F1 knockdown (K: siRNA-#1. L: siRNA-#2) (n = 6).
(M and N) Ubiquitination assay showing that the degree of p53 ubiquitination using BxPC-3 cells expressing the indicated plasmids.
(O) IB analysis with the indicated antibodies in control or $KRAS^{G12D}$-overexpreesed BxPC -3 cells with or without *Tp53* knockdown (siRNA-#2).
(P and Q) qRT-PCR assays show the mRNA expression of *UBE2T* in BxPC-3 cells with or without $KRAS^{G12D}$ expression and/or *Tp53* knockdown (P: siRNA-#1. Q: siRNA-#2) (n = 3).
(R) qRT-PCR assays show the mRNA expression of UBE2T and E2F1 in $KRAS^{G12D}$-overexpressed BxPC-3 cells with or without p53 expression and/or palbociclib treatment (n = 3).
(S) MST curve shows the interaction between Rb and E2F1. K_D, the equilibrium dissociation constant.
(T) qRT-PCR assays show the mRNA expression of UBE2T in $KRAS^{G12D}$-overexpressed BxPC-3 cells with or without E2F1 and/or p53 mutants overexpression (n = 3).
Mean ± SD, Student's t test. **P < 0.01, ns, not significant.

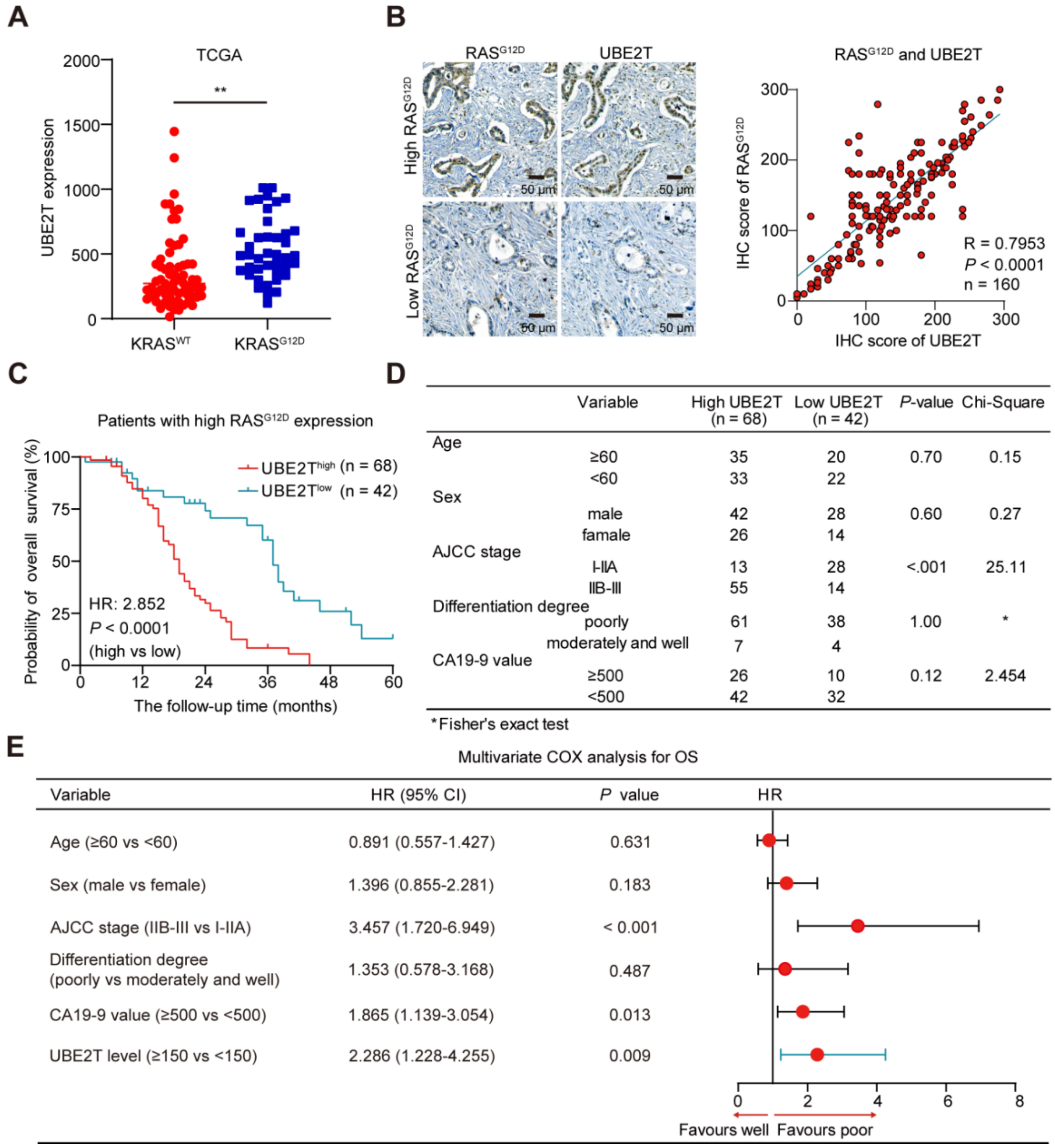

Figure S7. UBE2T level is negatively associated with prognosis in patients with KRAS^{G12D}-mutated PDAC. Related to Figure 5.

(A) The relative *UBE2T* mRNA expression level in patients with KRAS^{WT} or KRAS^{G12D} mutation from TCGA database.

(B) Representative images of RAS^{G12D} and UBE2T staining in PDAC tissues from patients (left). The protein level correlation between UBE2T and RAS^{G12D} in patients with PDAC from our cohort (n = 160) using linear regression analysis (right).

(C) Kaplan-Meier survival curves with log-rank test for patients stratified by UBE2T protein levels in patients with high RAS^{G12D} level (n = 110).

(D) The correlation between UBE2T level and age, sex, AJCC stage, differentiation degree, and CA19-9 value in patients with high RAS^{G12D} level (n = 110).

(E) Multivariate COX regression analysis of the overall survival in patients with high RAS^{G12D} level (n = 110). Bars indicate 95% confidence intervals.

Mean ± SD, Student's t test. **P < 0.01.

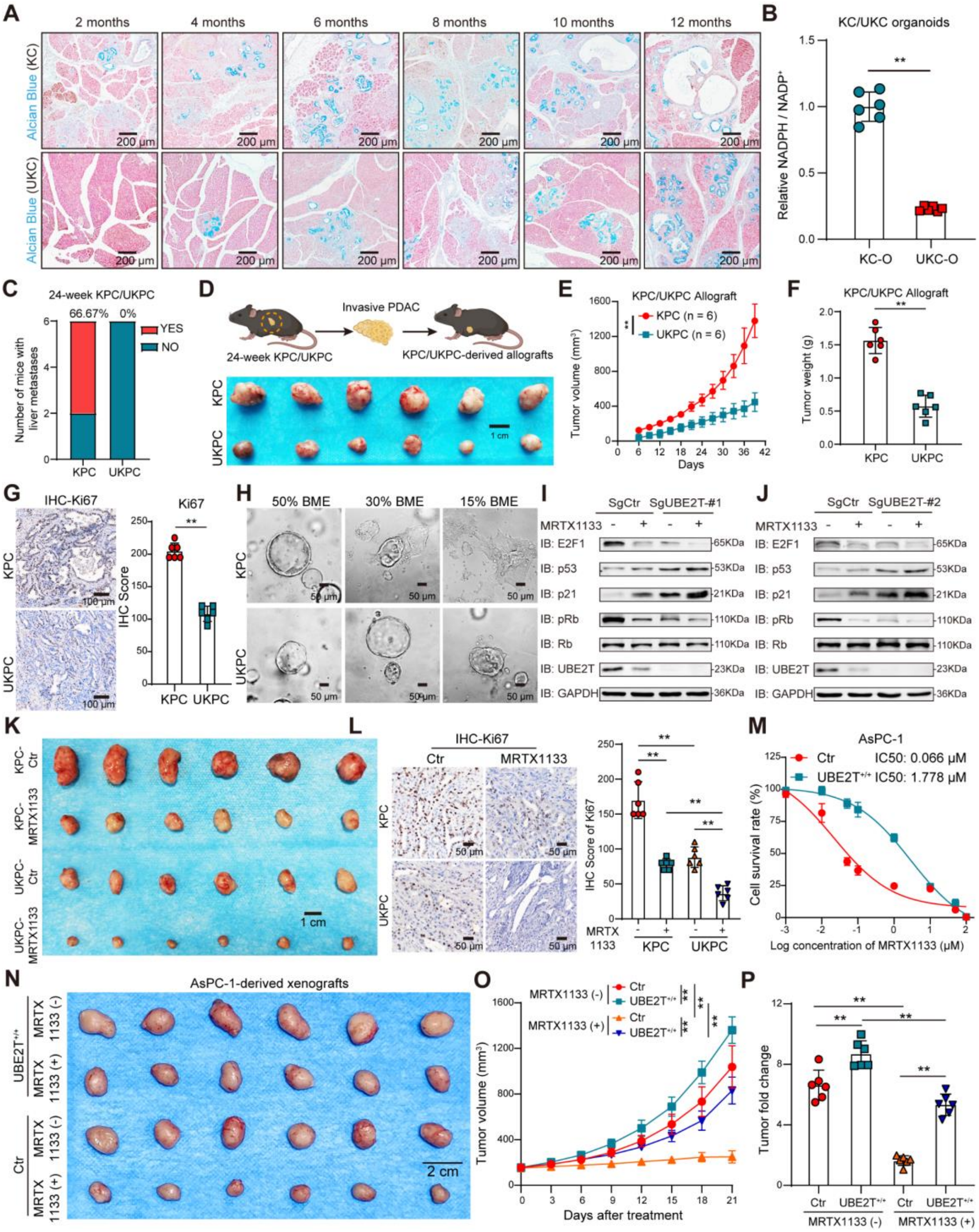

Figure S8. *UBE2T* deletion inhibits malignant progression and resistance to MRTX1133 in PDAC with KRAS^{G12D} mutation. Related to Figure 5.

(A) Representative images of pancreatic tissues stained with Alcian blue in 2/4/6/8/10/12-month-old KC or UKC mice.

(B) G6PD enzyme activity detection assessed by NADPH/NADP$^+$ in KC or UKC organoids (n = 6).

(C) Statistical analysis of liver metastasis rates in 24-week-old KPC and UKPC mice (n = 6).

(D-G) Representative images of tumor (D) and quantitation of tumor growth (E), tumor weight (F), and Ki67 level (G) in 24-week-old KPC and UKPC allografts (n = 6).

(H) Invasive status of KPC and UKPC organoids in specified concentrations of basement membrane extract.

(I and J) IB analysis with the indicated antibodies in SgCtr or SgUBE2T PDO-3 with or without MRTX1133 (10 μM) treatment (I: SgRNA-#1. J: SgRNA-#2).
(K and L) Representative images of tumor (J) and quantitation of Ki67 level (K) in KPC or UKPC allografts with or without MRTX1133 treatment (30 mg/kg/day) (n = 6).
(M) Sensitivity to MRTX1133 in control and UBE2T-overexpressed AsPC-1 cells (n = 6).
(N-P) Representative images of tumors (N) and quantitation of tumor growth (O) and fold change (P) in control and UBE2T-overexpressed AsPC-1 cell-derived xenografts treated with or without MRTX1133 (30 mg/kg/day) (n = 6).
Mean ± SD, Student's t test. **P < 0.01.

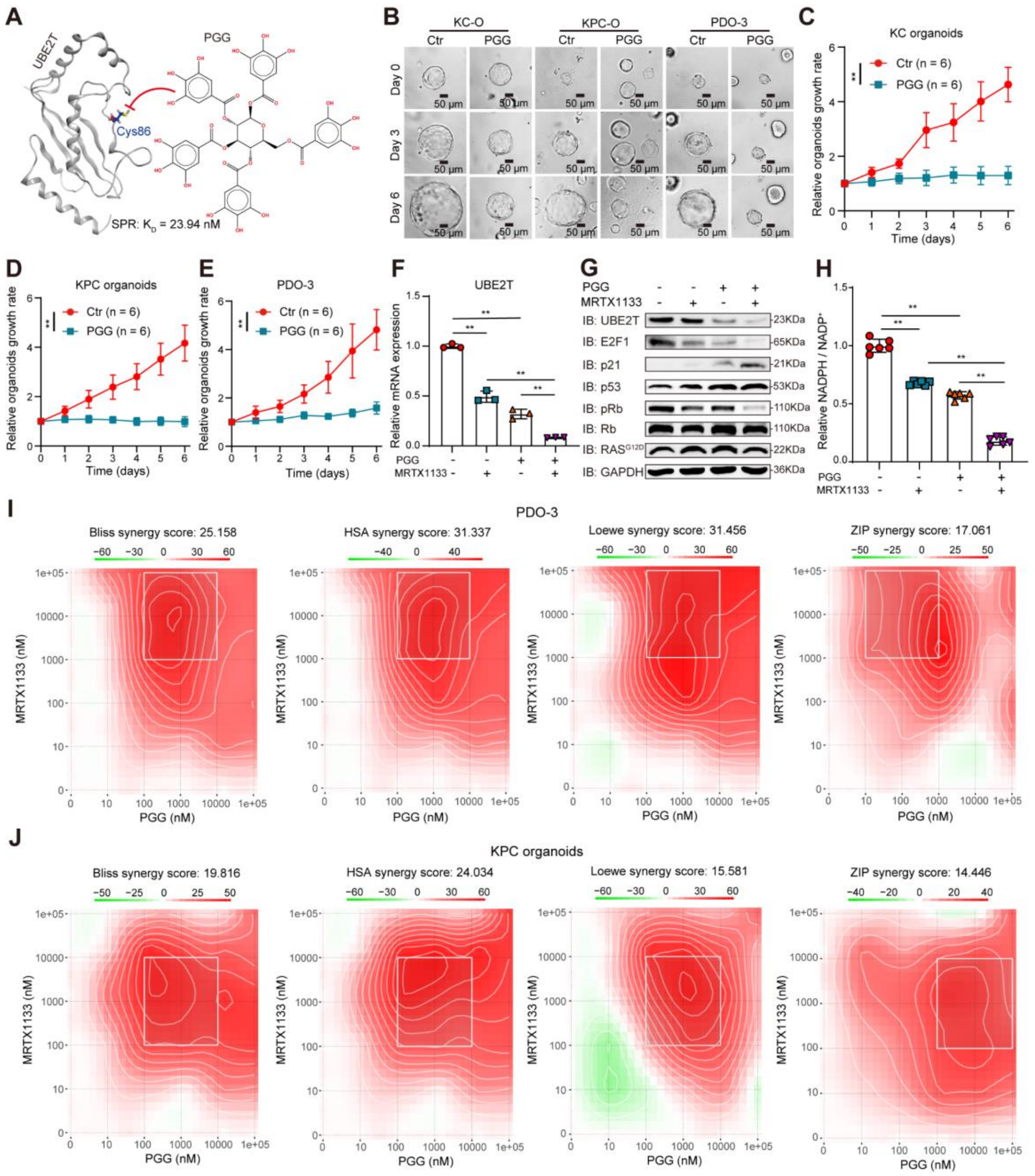

Figure S9. PGG inhibits progression and synergizes with MRTX1133 *in vitro*. Related to Figure 6.

(A) Schematic diagram of PGG targeting UBE2T.

(B-E) Representative images (B) and quantification of KC (C) and KPC-derived organoids (D), and PDO-3 (E) treated with PGG (10 μM, n = 6).

(F) qRT-PCR assay shows the mRNA expression of UBE2T in PDO-3 with or without PGG and/or MRTX1133 treatment (10 μM).

(G) IB analysis with the indicated antibodies in KPC-derived organoids with or without PGG (10 μM) and/or MRTX1133 (10 μM) treatment.

(H) G6PD enzyme activity measured by NADPH/NADP$^+$ ratio in KPC-derived organoids with or without PGG (10 μM) and/or MRTX1133 (10 μM) treatment (n = 6).

(I and J) Synergy analysis of MRTX1133 and PGG in PDO-3 (I) and KPC organoids (J).

Mean ± SD, Student's t test. **P < 0.01.

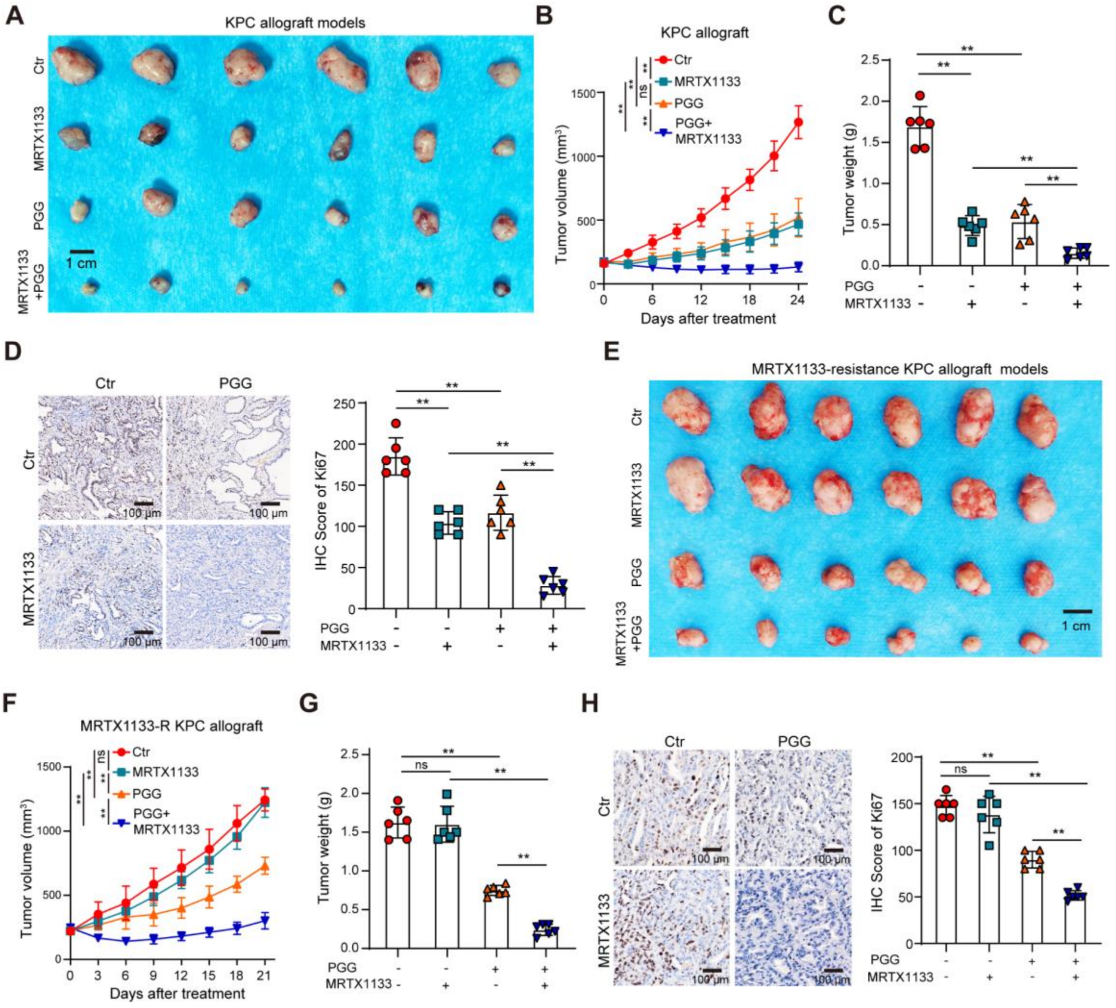

Figure S10. PGG synergizes with MRTX1133 *in vivo*. Related to Figure 6.

(A-D) Representative images of tumor (A), quantitation of tumor growth (B), tumor weight (C), and Ki67 level (D) in KPC allografts with or without PGG (40 mg/kg/day) and/or MRTX1133 treatment (30 mg/kg/day) (n = 6).

(E-H) Representative images of tumor (E), quantitation of tumor growth (F), tumor weight (G), and Ki67 level (H) in MRTX1133-resistance KPC allografts with or without PGG (40 mg/kg/day) and/or MRTX1133 treatment (30 mg/kg/day) (n = 6).

Mean ± SD, Student's t test. **P < 0.01, ns, not significant.

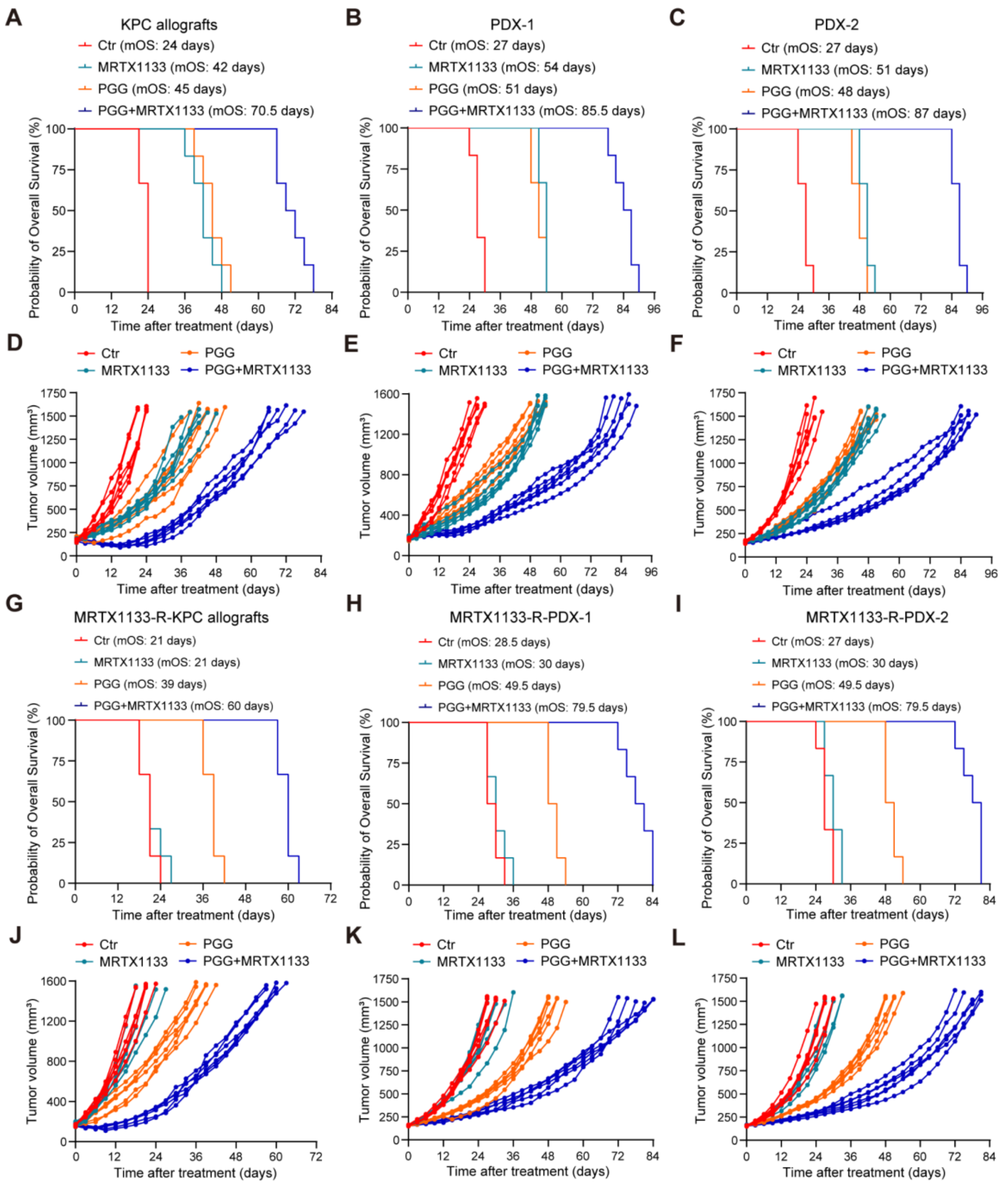

Figure S11. The combination of PGG and MRTX1133 prolongs the overall survival of PDAC with KRAS^{G12D} mutation. Related to Figure 6.

(A-C) Overall survival of KPC allografts (A), PDX-1 (B), and PDX-2 (C) models treated with PGG (40 mg/kg/day) and/or MRTX1133(30 mg/kg/day) (n ≥ 6).

(D-F) Tumor growth of KPC allografts (D), PDX-1 (E), and PDX-2 (F) models treated with PGG (40 mg/kg/day) and/or MRTX1133(30 mg/kg/day) (n ≥ 6).

(G-I) Overall survival of MRTX1133-resistance KPC allografts (G), PDX-1 (H), and PDX-2 (I) models treated with PGG (40 mg/kg/day) and/or MRTX1133(30 mg/kg/day) (n ≥ 6).

(J-L) Tumor growth of MRTX1133-resistance KPC allografts (J), PDX-1 (K), and PDX-2 (L) models treated with PGG (40 mg/kg/day) and/or MRTX1133(30 mg/kg/day) (n ≥ 6).

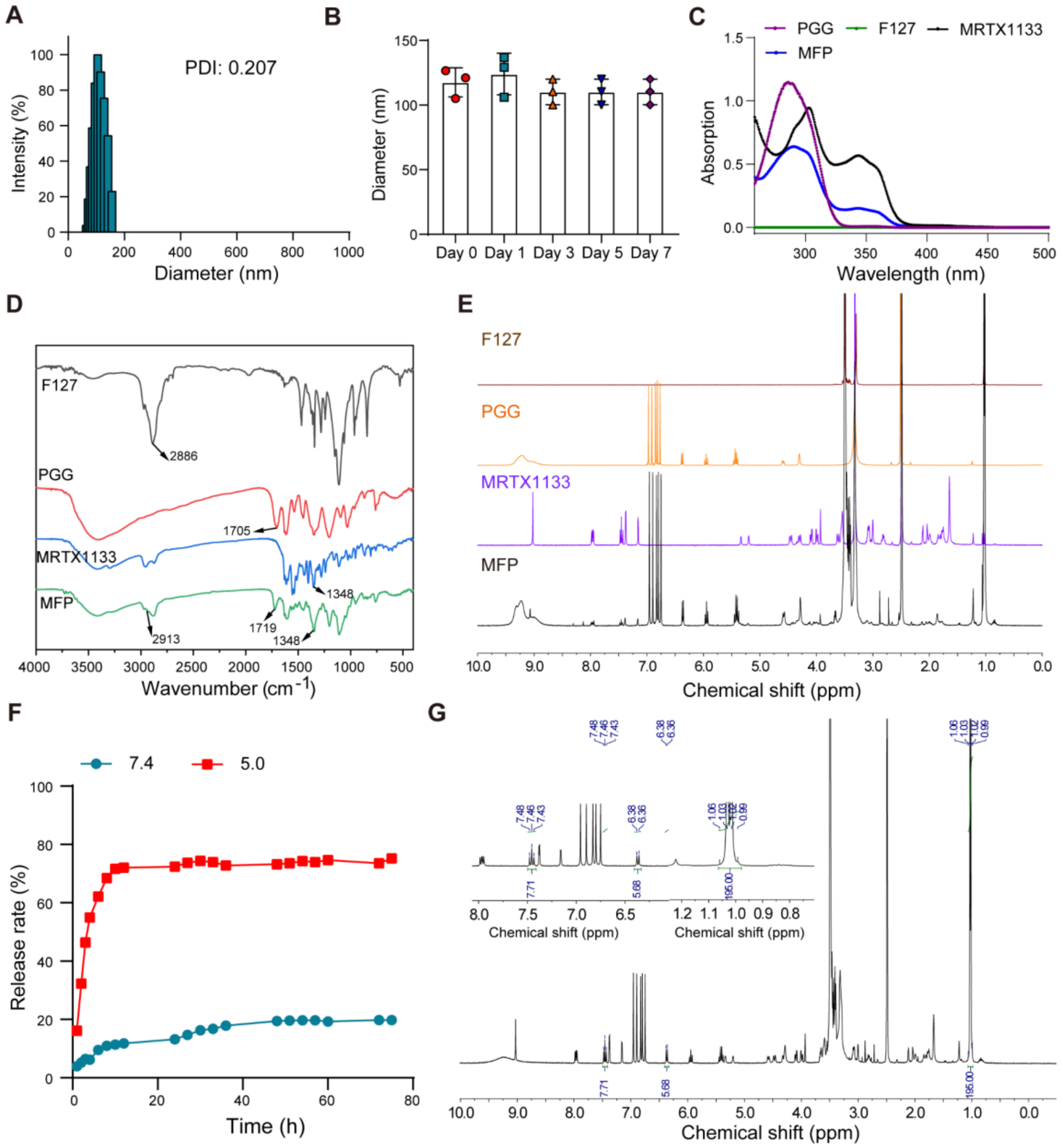

Figure S12. Characterization of the MFP nano-delivery system. Related to Figure 7.

(A) Hydrodynamic diameter of MFP examined at pH 7.4 buffer.

(B) Diameter of MFP examined in Day0/1/3/5/7(n = 3).

(C) UV-vis spectrum of F127, PGG, MRTX1133 and MFP in DMSO.

(D) FT-IR spectra of F127, PGG, MRTX1133 and MFP.

(E) Superimposed 1HNMRspectra of F127, PGG, MRTX1133 and MFP in DMSO-d6.

(F) MRTX113 release profiles of MFP examined at pH 7.4 PBS and 5.0 PBS.

(G) The HNMR spectrum of MFP and its integral area of characteristic proton signal.

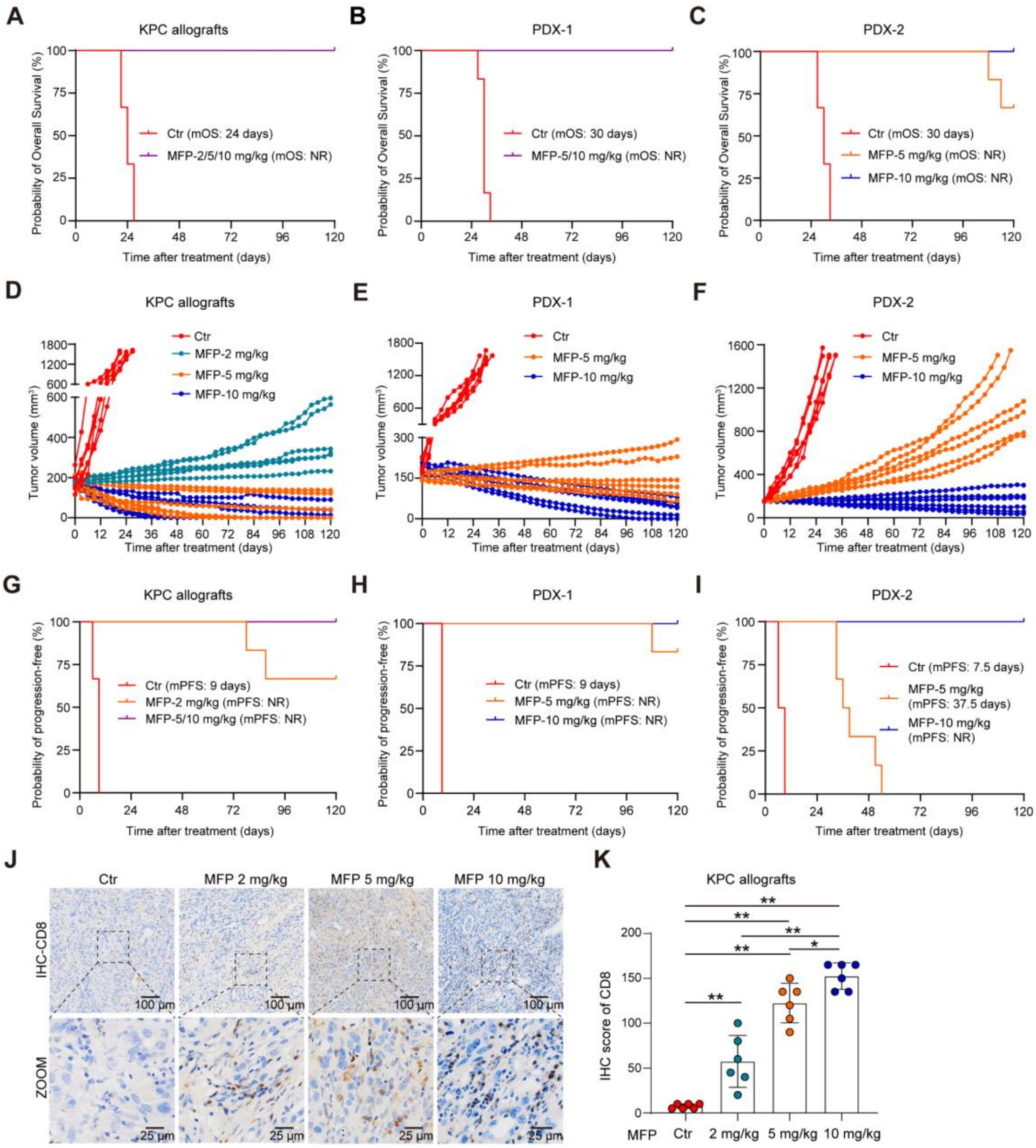

Figure S13. MFP reduces tumor volume and sustains long-term survival in PDAC. Related to Figure 7.

(A-C) Overall survival of KPC allografts (A), PDX-1 (B), and PDX-2 (C) models treated with 2 mg/kg/day, 5 mg/kg/day or 10 mg/kg/day MFP (n ≥ 6). NR, not reached.

(D-F) Tumor growth of KPC allografts (D), PDX-1 (E), and PDX-2 (F) models treated with 2 mg/kg/day, 5 mg/kg/day and 10 mg/kg/day MFP (n ≥ 6).

(G-I) Progression-free survival (PFS) of KPC allografts (G), PDX-1 (H), and PDX-2 (I) models treated with 2 mg/kg/day, 5 mg/kg/day and 10 mg/kg/day MFP (n ≥ 6).

(J and K) Representative IHC images (J) and quantitation (K) of CD8 in KPC allografts treated with 2 mg/kg/day, 5 mg/kg/day or 10 mg/kg/day MFP (n = 6).

Mean ± SD, Student's t test. *P < 0.05. **P < 0.01.

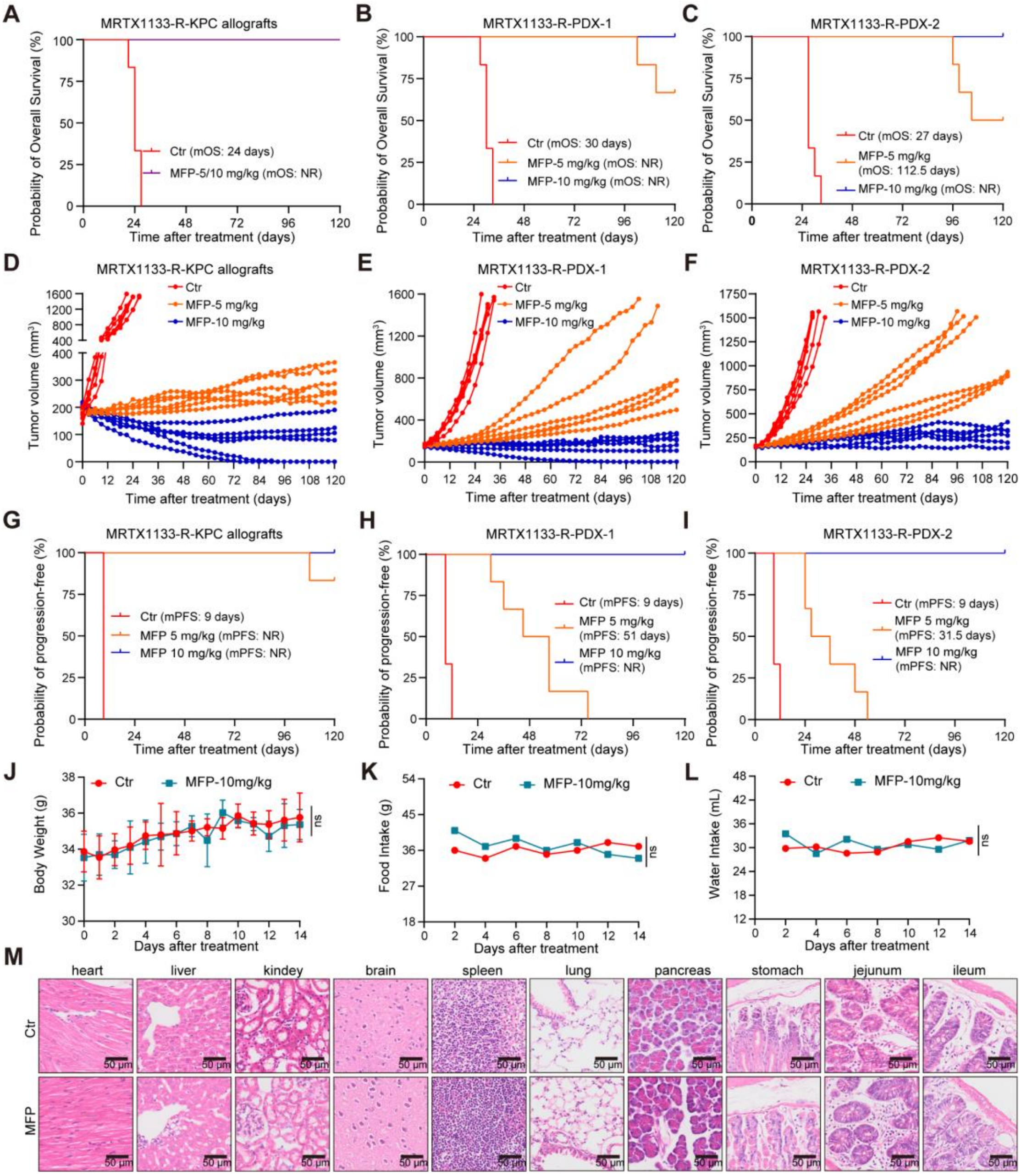

Figure S14. Efficacy and acute toxicity evaluation of MFP treatment. Related to Figure 7.

(A-C) Overall survival of MRTX1133-resistance KPC allografts (A), PDX-1 (B), and PDX-2 (C) models treated with 5 mg/kg/day and 10 mg/kg/day MFP (n ≥ 6). NR, not reached.

(D-F) Tumor growth of MRTX1133-resistance KPC allografts (D), PDX-1 (E), and PDX-2 (F) models treated with 5 mg/kg/day and 10 mg/kg/day MFP (n ≥ 6).

(G-I) PFS of MRTX1133-resistance KPC allografts (G), PDX-1 (H), and PDX-2 (I) models treated with 5 mg/kg/day and 10 mg/kg/day MFP (n ≥ 6).

(J-L) body weight (J), food intake (K), and water intake (L) in Kunming mice treated with or without MFP (10 mg/kg) (n = 3).

(M) Representative H&E images of the indicated visceral organs in Kunming Mouse treated with or without MFP (10 mg/kg).

Mean ± SD, Student's t test. ns, not significant.
